# Muscle synergies and functional network organization underlying Tai Chi movements: a pilot cross-Sectional study towards a personalized Neuromechanical Baseline

**DOI:** 10.3389/fbioe.2026.1928241

**Published:** 2026-09-18

**Authors:** Zhi Li, Xiupan Wei, Ting Zhou, Xueming Fu, Qian Wang, Wenhao Zou, Pengyi Gao, Pei Wang, Shusen He, Zhe Yang, Aiguo Song, Hongxing Wang

**Affiliations:** 1 Department of Rehabilitation Medicine, Southeast University Zhongda Hospital, Nanjing, China; 2 Department of Rehabilitation Medicine, School of Medicine, Sichuan Provincial People’s Hospital, University of Electronic Science and Technology of China, Chengdu, China; 3 School of Biomedical Engineering, University of Science and Technology of China, Hefei, China; 4 Department of Rehabilitation Medicine, Qingyun Traditional Chinese Medicine Hospital, Dezhou, China; 5 The Intelligent Information Processing Laboratory, Southeast University, Nanjing, China

**Keywords:** functional network, muscle activation patterns, muscle synergy, surface electromyography, Tai Chi

## Abstract

Tai Chi (TC) integrates coordinated upper–lower limb movements and bilateral motor patterns that enhance neuromuscular control. Despite its rehabilitation potential, patients with motor dysfunction often find standard TC postures difficult to perform, and target muscles vary across individuals. This study aimed to characterize muscle synergies and functional networks during TC to inform personalized rehabilitation. Twenty-three experienced practitioners performed the 24-form TC sequence while surface electromyography (sEMG) signals were recorded from 16 bilateral upper- and lower-limb muscles. Muscle synergies were extracted using non-negative matrix factorization (NNMF), with model order selected using variance accounted for (VAF). Normalized mutual information (NMI) quantified intermuscular connectivity, and graph-theoretical metrics - clustering coefficient (*C*), path length (*L*), modularity (*Q*), and small-worldness (σ = γ/λ) - characterized network organization and hub muscles. Two whole-body synergies were identified. They showed high repeatability across trials (cosine similarity = 0.9708 ± 0.0352) and high consistency across participants (0.9281 ± 0.049). In the 23-participant NMI subset, connections between opposite sides within the upper or lower limbs were stronger than connections on the same side (mean paired ΔNMI = 0.0332, 95% bootstrap CI [0.0322, 0.0342], Holm-adjusted p = 0.0059). In the descriptive ranking analysis, 88.3% of the five highest-NMI pairs in each movement connected opposite sides of the body. The functional networks showed small-world properties. Modularity ranged from 0.23 to 0.41, and 62.5% of movements had Q > 0.30. Movements with Q ≤ 0.30 were interpreted as having weaker community structure. Lower-limb muscles, especially the gastrocnemius and tibialis anterior, frequently acted as network hubs. This pilot study demonstrates that TC elicits stable bilateral muscle synergies and small-world network organization in experienced practitioners, indicating an underlying neuromotor framework for coordinated movement. Future studies should examine whether these synergy patterns can be adapted or retrained in patients with motor impairments, such as stroke survivors, using appropriate control groups.

**Clinical Trial Registration:**

www.chictr.org.cn, identifier ChiCTR2400080158.

## 1 Introduction

Tai Chi (TC) boasts a long history and embodies Chinese traditional medical and fitness culture, serving as a tangible application of the Chinese medicine philosophy of “disease prevention and treatment” (Qin et al., 2020). By integrating specific postures, breathing techniques, and meditation, TC is associated with improvements in general health status and functional performance across diverse populations (Song et al., 2020). Owing to its efficacy in promoting overall health and managing age-related conditions, the clinical application of TC has expanded globally, and it is frequently recommended for older adults and patients with chronic diseases to ameliorate their symptoms (Wang et al., 2022). This integrative brain-body perspective is consistent with the emerging health-neuroscience framework, which treats TC as a behaviorally mediated intervention linking neural regulation, bodily function, and health outcomes (Tang and Tang, 2024).

Previous studies have demonstrated that TC can significantly enhance rehabilitation outcomes in patients with motor dysfunction (Zhao et al., 2022). However, TC still encounters significant challenges in clinical practice. Due to patients’ inherent motor impairments, many are unable to perform the complex, standardized TC routines (Guo et al., 2023; Feng et al., 2023). Existing adaptations, such as seated training (Zhao et al., 2022) or weight-supported systems (Huang et al., 2019), have indeed eased access to TC practice. However, these modifications may disrupt the intrinsic neuromuscular coordination patterns inherent to traditional Tai Chi, thereby potentially modifying its biomechanical behavior.

Research indicates that, compared with ordinary walking, TC may more effectively improve hip range of motion and enhance neuromuscular coordination while operating under safer conditions (Duan et al., 2020). Kinematic analyses further show that TC gait imposes greater balance challenges and requires increased muscular engagement of the lower limbs. To meet these demands, individuals appear to develop adaptive neuromuscular control strategies that help preserve stability and reduce fall risk (Yang and Liu, 2020), Consistent with this, long-term TC practitioners demonstrate faster and more precise neuromuscular responses to perturbations—for example, shortened gastrocnemius response times and a reduced incidence of antagonist co-contraction during slip recovery (Gatts, 2008; Gatts and Woollacott, 2006) — indicating superior neuromuscular control and reaction speed. The resulting improvements in neuromuscular efficiency and attenuation of postural sway are associated with better performance on sit-to-stand tasks (Mozhu and Jiayu, 2025), and the overall enhancement in coordination contributes to greater movement stability (Zhang et al., 2025). Taken together, the whole-body coordination patterns elicited by TC could help establish a quantitative biomechanical reference framework to inform studies of neuromuscular impairment in clinical populations. Recent work in elite Tai Chi athletes further demonstrates that visually symmetric lower-limb tasks can retain similar synergy composition while showing leg-specific activation timing, underscoring the influence of training history and functional limb roles (Mo and Jing, 2025).

Surface electromyography (sEMG) is a non-invasive technique for recording the bioelectric potentials generated by skeletal muscles during contraction, thereby enabling detailed analysis of their electrophysiological properties (Merletti et al., 2001). Recent studies have applied sEMG to the movement analysis of TC (Chan et al., 2003). The theory of muscle synergy (MS) posits that MS represents the fundamental unit by which the central nervous system (CNS) controls the musculoskeletal system to execute complex movements (Ghislie et al., 2020). Cheung et al. further demonstrated that motor cortical areas primarily select and activate a fixed set of spinal and brainstem muscle synergies, and that in stroke patients, synergy composition is largely preserved between the affected and unaffected limbs. Nevertheless, nonnegative matrix factorization (NMF) of EMG signals has revealed three post-stroke synergy coordination patterns—preservation, merging, and fractionation—that vary with impairment severity and time since stroke; these patterns correlate with motor deficits and can serve as physiological markers to guide rehabilitation by targeting synergies with altered activation (Cheung et al., 2009; Cheung et al., 2012). This framework has been widely applied in pathological gait analysis, motor function assessment, and rehabilitation therapy. Mutual information (MI) is a statistical measure capable of capturing both linear and nonlinear dependencies between time series. When two signals are completely independent, their MI value is zero (Jeong et al., 2001). MI has been widely utilized in constructing functional connectivity networks based on sEMG signals (Junquera-Godoy et al., 2024; Madeleine et al., 2016). For instance, normalized mutual information (NMI) can reveal dynamic interactions among co-activated muscles, offering insights into intermuscular functional connectivity (Cholewicki et al., 1997).

This study aims to build and analyze muscle functional networks and synergy patterns across all 24 TC forms to uncover their underlying neuromuscular coordination mechanisms. By mapping key muscle connections and network topologies, the resulting atlas provides a quantitative biomechanical reference for understanding neuromuscular coordination in Tai Chi and may facilitate future rehabilitation research in patients with stroke.

## 2 Materials and methods

### 2.1 Sample population

The study recruited 23 healthy participants (12 females and 11 males; age: 20.48 ± 1.53 years; height: 170.48 ± 8.41 cm; weight: 64.48 ± 9.84 kg). All participants were in good health, had prior TC practice experience sufficient to perform the 24-form sequence independently, and reported no lower limb or joint injuries in the past 6 months. Based on their preferred limb when initiating gait, all participants were right-leg dominant (Niu et al., 2011). None exhibited neurological impairments or structural abnormalities that could interfere with the experiment. All participants provided written informed consent, and all procedures were conducted in accordance with the Declaration of Helsinki. Participant characteristics are detailed in Table 1.

**TABLE 1:** Basic information of participants.

Participants	Gender	Age (y)	Body height (cm)	Body weight (kg)	Training experience (y)
1	m	20	172	86	1–3
2	m	20	180	70	>3
3	f	19	167	57	<1
4	f	22	156	50	1–3
5	f	19	164	55	1–3
6	f	20	166	72	<1
7	f	21	175	57	<1
8	f	19	156	65	<1
9	m	21	189	81	1–3
10	m	19	171	62	>3
11	m	20	168	68	1–3
12	f	21	174	56	1–3
13	m	19	183	67	>3
14	m	24	170	75	>3
15	m	24	183	78	>3
16	m	18	170	70	<1
17	m	20	172	70	1–3
18	f	22	162	55	>3
19	f	19	165	56	1–3
20	f	21	168	55	1–3
21	m	20	179	71	>3
22	f	22	158	52	1–3
23	f	21	173	55	1–3

m/f, male/female; y, years; cm, centimeter; kg, kilogram. Training duration was collected only as categorical bands (<1, 1–3, or >3 years); weekly practice frequency was not recorded.

### 2.2 Trial protocol

Participants performed the 24 forms as separate trials in the standard sequence rather than as one uninterrupted routine. For each form, the final posture of the preceding form served as the starting position. Before data acquisition, each participant completed a preliminary practice session comprising all 24 forms to become familiar with the experimental procedures and minimize errors during formal testing. An investigator’s verbal start cue marked movement initiation and simultaneously triggered sEMG recording; the stop cue was given after the participant reached the final posture and terminated the recording. Each form was repeated three times before proceeding to the next form, yielding 72 trials per participant. A 1-min rest interval was provided between repetitions to minimize muscle fatigue.

### 2.3 Data acquisitions

sEMG signals were recorded throughout the experimental procedure using a surface EMG analysis and feedback system (SG-1600B, Dno Medical Technology Co., Ltd., Zhejiang, China), A total of 16 muscles were monitored:

Upper limb muscles (8): left deltoid (DEL_L), right deltoid (DEL_R), left biceps brachii (BB_L), right biceps brachii (BB_R), left triceps brachii (TB_L), right triceps brachii (TB_R), left extensor carpi radialis longus (ECRL_L), and right extensor carpi radialis longus (ECRL_R).

Lower limb muscles (8): left rectus femoris (RF_L), right rectus femoris (RF_R), left biceps femoris (BF_L), right biceps femoris (BF_R), left gastrocnemius (GC_L), right gastrocnemius (GC_R), left tibialis anterior (TA_L), and right tibialis anterior (TA_R). Electrode placement is illustrated in Figure 1a.

**FIGURE 1:**
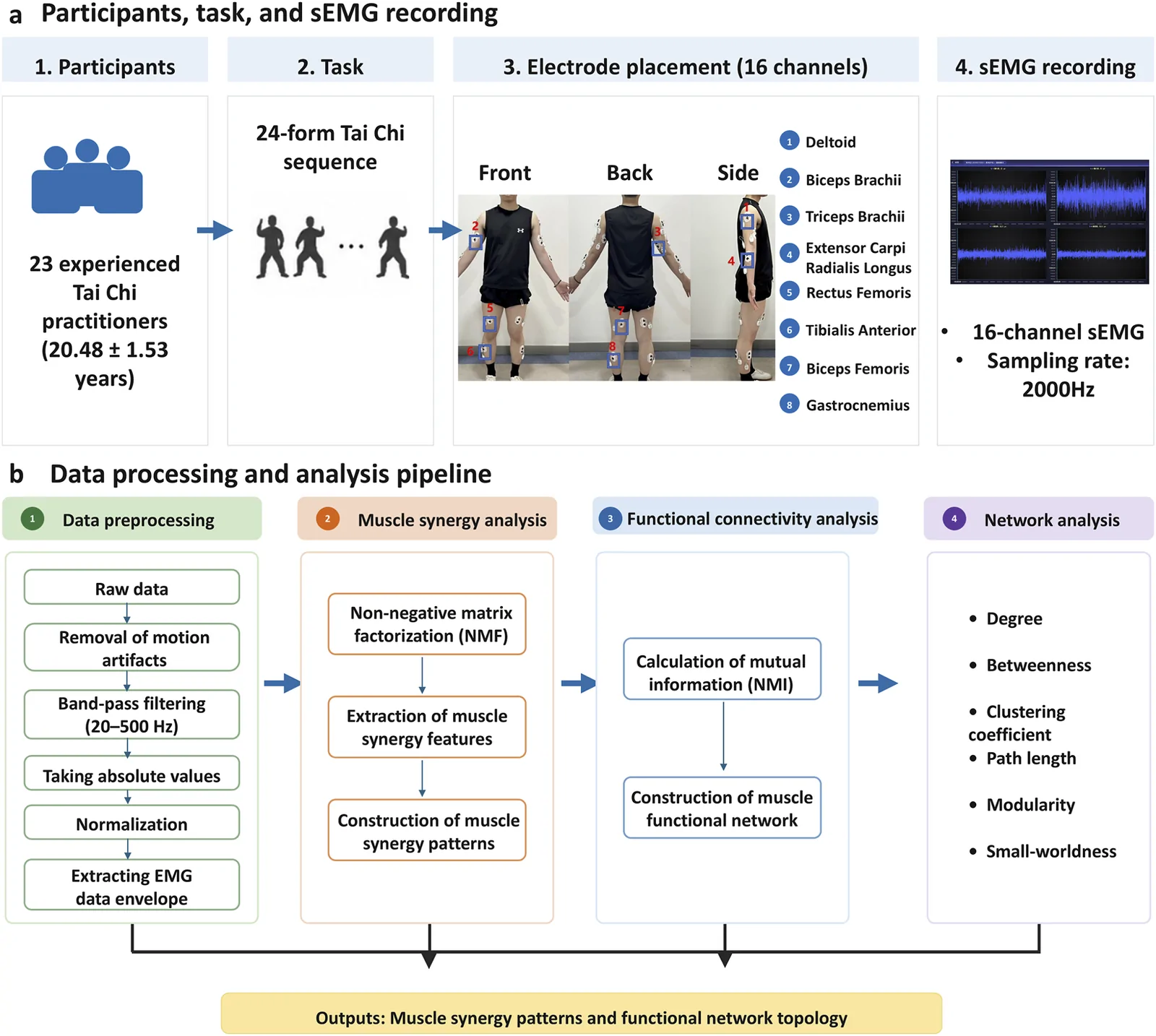
Overview of sEMG acquisition and preprocessing. **(a)** Electrode placement over 16 bilateral upper- and lower-limb muscles: deltoid (DEL), biceps brachii (BB), triceps brachii (TB), extensor carpi radialis longus (ECRL), rectus femoris (RF), biceps femoris (BF), gastrocnemius (GC), and tibialis anterior (TA). L and R indicate the left and right sides, respectively. **(b)** Preprocessing and analysis workflow, including band-pass filtering (20–500 Hz), full-wave rectification, global-maximum amplitude normalization, and subsequent muscle-synergy or normalized mutual information (NMI) analysis.

The sEMG signals were sampled at 2000 Hz. Upon completion of data collection, the signals were labeled according to the TC movement and subject ID, then imported into MATLAB® (R2022a, The MathWorks Inc., Natick, MA, USA) for offline analysis using custom-developed scripts.

### 2.4 Data Processing

The raw sEMG signals were imported into MATLAB 2022a for preprocessing. A band-pass filter (20–500 Hz) was applied to remove motion artifacts and retain the valid frequency range of muscle activity. The filtered signals were then full-wave rectified to obtain non-negative values, and subsequently normalized to their global maximum amplitude to generate standardized sEMG envelopes (Ghislieri et al., 2020). These normalized envelopes were used for muscle synergy extraction and NMI computation. The preprocessing pipeline is illustrated in Figure 1b. Normalization was amplitude-based rather than time-based: each full-wave-rectified signal was scaled to its global maximum amplitude, producing a unitless envelope. The acquired time axis was retained, and no temporal interpolation or cycle rescaling was applied before NNMF or NMI analysis.

### 2.5 Muscle synergy extraction

#### 2.5.1 Non-negative matrix factorization (NNMF)

Surface EMG (sEMG) envelopes were preprocessed and normalized before decomposition (Lee and Seung, 1999). Muscle synergies were extracted using non-negative matrix factorization (NNMF), which approximates the original data matrix V (Taborri et al., 2018).

V≈W·H

Here, V is an m×n matrix (where m is the number of muscle channels, and n is the number of time samples). The activation coefficients H describe the temporal modulation of sEMG (the temporal component of motor control), while the weight vectors W describe the spatial contributions of the synergies (the spatial component of motor control) (Ghislieri et al., 2020). Results are returned once the maximum iteration count is reached or convergence occurs.

W and H were learned by iterative multiplicative updates to minimize the reconstruction error $∥X-WH{∥}_{F}$ , where $∥\cdot {∥}_{F}$ : denotes the Frobenius norm. Convergence was declared when the relative change in error fell below tolerance or a maximum iteration count was reached. To select the number of synergies k we used Variance Accounted For (VAF), defined as:

$$\text{VAF}=1-\frac{{\left\|V-WH\right\|}_{F}^{2}}{{\left\|V\right\|}_{F}^{2}}$$

and adopted a conservative criterion of VAF≥0.90 for the full 16-channel dataset and for anatomically partitioned submatrices (left/right, upper/lower limb). If a submatrix failed to reach VAF≥0.90 with the global k (Clark et al., 2010; Barradas et al., 2020). The modal value of k across participants and sessions was taken as the group-level synergy number (Ghislieri et al., 2020). Model order was determined separately for the complete 16-channel whole-body matrix and the laterality- and limb-specific submatrices. For each matrix, k was increased from one to 5, and the smallest k yielding a VAF of ≥0.90 was retained. The increase in VAF obtained by adding the next synergy was also examined as an index of diminishing improvement, with an increment of <0.05 indicating limited additional reconstruction benefit. The left-, right-, upper-limb-, and lower-limb submatrices were decomposed independently to assess whether regional muscle activation patterns could be adequately reconstructed. Thus, the model order selected for a regional submatrix was specific to that partition and was not used to redefine the number of synergies extracted from the complete whole-body matrix.

Because NNMF returns synergies in arbitrary order across trials and subjects, synergy vectors were reordered prior to averaging using cosine similarity (Li YA. et al., 2023) and cosine similarity quantifies the similarity between two vectors by normalizing their dot product with the product of their magnitudes (Oliveira et al., 2014):

$$\text{Cosine Similarity}=\frac{A\cdot B}{\left\|A\right\|\left\|B\right\|}$$

For two vectors A and B , A·B represents their dot product, while $\left\|A\right\|$ and $\left\|B\right\|$ denote the norms of the respective vectors. Cosine similarity values close to 0 and one indicate dissimilarity and high similarity, respectively. Based on similar studies, a cosine similarity value exceeding 0.684 is considered indicative of similarity (Oshima et al., 2022). Group-average weight sets were then compared back to individuals to verify representativeness (Kibushi et al., 2018).

### 2.6 Normalized mutual Information (NMI)

To quantify pairwise interactions between muscles, we computed normalized mutual information (NMI) between every muscle pair based on their sEMG time series. Probability distributions were estimated using histograms with small regularization ϵ added to prevent numerical singularities. The Shannon entropy of a signal X with binned probabilities $P_{X}\left(i\right)$ is:

$$H\left(X\right)=-\sum_{i}P_{X}\left(i\right)\log\left(P_{X}\left(i\right)+\epsilon \right)$$

$$H\left(Y\right)=-\sum_{j}P_{Y}\left(j\right)\log\left(P_{Y}\left(j\right)+\epsilon \right)$$

Joint entropy $H\left(X,Y\right)$ is defined analogously from the joint histogram $P_{XY}\left(i,j\right)$ . Mutual information (MI) between channels X and Y is:

$$I\left(X;Y\right)=\sum_{i}\sum_{j}P_{XY}\left(i,j\right)\log\frac{P_{XY}\left(i,j\right)}{P_{X}\left(i\right)P_{Y}\left(j\right)}$$

MI is non-negative and bounded above by $\min\left\{H\left(X\right),H\left(Y\right)\right\}$ . To obtain a normalized measure in [0,1] suitable for between-subject comparison we used:

$$\text{NMI}\left(X,Y\right)=\frac{I\left(X;Y\right)}{\min\left(H\left(X\right),H\left(Y\right)\right)}$$

For each participant and motion condition, NMIs were averaged across three repeats; group matrices were produced by averaging participant NMIs. With 16 muscles there are 120 unique pairs (upper triangular), yielding a symmetric 16 × 16 NMI matrix per condition. All three repetitions contributed equally. Thresholding was performed after participant-level NMI matrices had been averaged; consequently, one movement-specific threshold was applied to each group-level matrix rather than being adjusted for individual participants.

### 2.7 Functional network construction and graph metrics

The group-averaged NMI matrix served as the weighted connectivity matrix. For each movement, candidate thresholds T from 0.00 to 1.00 were scanned in increments of 0.01, and edges with NMI ≥ T were retained in a binary undirected graph. A candidate graph was admissible only if all 16 nodes belonged to one connected component, no node was isolated, and the mean degree exceeded 2ln (16) = 5.545. To maximize sparsity while preserving connectivity, the largest threshold satisfying all three constraints was selected for that movement. Thresholds were therefore movement-specific but not participant-specific. Small-world metrics were calculated only after threshold selection and were not used to tune T, avoiding circular selection (Chen L. et al., 2021; Goldman et al., 2002; Hui et al., 2025).

Node $k_{i}$ degree and network average degree $\langle k\rangle$ were computed as:

$$k_{i}=\sum_{j}A_{ij},\langle k\rangle =\frac{1}{n}\sum_{i=1}^{n}k_{i}$$

with N the number of nodes. Betweenness centrality $C_{B}\left(v\right)$ for node v was computed as

$$C_{B}\left(v\right)=\sum_{s\neq v\neq t}\frac{\sigma_{st}\left(v\right)}{\sigma_{st}}$$

where $\sigma_{st}$ is the number of shortest paths between nodes s and t and $\sigma_{st}\left(v\right)$ those passing through v . Nodes with both high degree and high betweenness centrality were labeled as movement-specific key muscles (Wang et al., 2016).

Global segregation and integration were quantified via clustering coefficient and path length. For node i , the clustering coefficient is (Wenhart et al., 2019; Dang et al., 2023):

$$C_{i}=\frac{2E}{k_{i}\left(k_{i}-1\right)}$$

where $E_{i}$ is the number of edges among i ’s neighbors. The network average clustering is:

$$C=\frac{1}{n}\sum_{i=1}^{n}C_{i}$$

Average shortest-path length L (global integration) is:

$$L=\frac{1}{n\left(n-1\right)}\sum_{i\neq j}\frac{1}{D\left(i,j\right)}$$

with $D_{ij}$ the shortest path length between i and j .

Small-world metrics were computed by comparison to equivalent random networks (preserving degree or weight distribution): $\gamma =C/C_{rand}$ , $\lambda =L/L_{rand}$ , and small-worldness σ=γ/λ . A network was considered small-world if γ>1 , λ≈1 , and σ>1 (Wenhart et al., 2019).

Modularity Q (Louvain-type partitioning; resolution = 1.0) quantified community structure: (Blondel et al., 2008; Lambiotte et al., 2008).

$$Q=\frac{1}{2m}\sum_{i,j}\left(A_{ij}-\frac{k_{i}k_{j}}{2m}\right)\delta \left(c_{i},c_{j}\right)$$

where m is total edge weight, $c_{i}$ the community index of node i , and δ the Kronecker delta. Higher Q indicates stronger community structure (values >0.3 typically denote meaningful modules). To stabilize estimates we randomized node orders and repeated modularity optimization; weighted networks were used throughout.

NNMF, NMI, and network analyses were performed in MATLAB and Python, with additional statistical tests conducted in Python 3.13.9. Participant-level NMI matrices (n = 23), each averaged across three repetitions, were treated as independent observations. For each participant, NMI values across 24 movements were averaged within four predefined connection groups: bilateral upper-limb, ipsilateral upper-limb, bilateral lower-limb, and ipsilateral lower-limb connections. Three exact two-sided sign-flip tests evaluated laterality, limb region, and their interaction using all possible sign patterns. Holm correction was applied, and 95% confidence intervals were estimated using 50,000 participant-level bootstrap samples. 120 muscle pairs were assessed using 100,000 within-participant permutations of the 16 muscle labels. Two-sided p values were adjusted using the Benjamini–Hochberg false-discovery rate at α = 0.05. These post hoc analyses were exploratory and assessed whether pairwise NMI differed from expectations after anatomical-label randomization. They did not test independence between the original sEMG signals because only repetition-averaged NMI matrices were available. Therefore, low or nonsignificant NMI was not interpreted as physiological separation. NNMF weights were not tested against zero because their non-negative constraint makes a two-sided zero test inappropriate. Further details are provided in Supplementary Material 1.

### 2.8 Equity, Diversity and inclusion statement

This laboratory mechanistic study recruited a convenience sample of 23 volunteer, experienced Tai Chi practitioners to obtain high-quality, standardized movement and sEMG recordings. Recruitment focused on participants with sufficient training to perform the 24-form sequence reliably; it was not designed to achieve representative sampling across race/ethnicity, socioeconomic status, geographic region, or clinical subgroups. Because of the small, specialist sample, we acknowledge limited generalisability to broader or marginalized populations and report this as a study limitation.

The investigator and author team is multidisciplinary and includes senior and early-career researchers with expertise in clinical rehabilitation, biomechanics, neurophysiology, and data analysis. We sought contributions from clinicians and methodologists to ensure clinical relevance and technical rigor. We do not disclose individual personal identities; however, the team structure intentionally combines clinical, engineering and rehabilitation perspectives to reduce single-discipline bias in study design and interpretation.

Data collection procedures were planned to be inclusive within the constraints of a laboratory study: all participants received plain-language study information and provided informed consent; sensor placement and experimental timing were adjusted to maximise participant comfort; rest breaks were provided and scheduling was flexible to accommodate participants’ availability. Because all participants were experienced practitioners and fluency in study instructions was required, the protocol did not require translation services or additional accessibility adjustments; such needs will be explicitly addressed in future clinical/translational phases.

In analysis and interpretation we accounted for sex as a potential confounding factor when feasible; no other equity-focused subgroup analyses (e.g., by ethnicity, socioeconomic status, or age) were possible given the small specialist sample. Participant characteristics are reported so readers can assess applicability. For future translational and clinical phases we commit to recruiting more diverse and representative cohorts, prespecifying subgroup and equity analyses, and engaging patient and public partners from under-represented communities to inform study design and dissemination.

## 3 Results

### 3.1 Two Synergies reconstructed whole-body muscle activity

The Variance Accounted For (VAF) quantifies the extent to which the shape and magnitude of the EMG matrix are preserved in the reconstruction based on NNMF (Afzal et al., 2022). Figure 2a shows the average VAF values for different numbers of muscle synergies, ranging from one to 5. As illustrated in Figure 2A, VAF increases with the number of extracted synergies. When two muscle synergies are used, the VAF exceeds 90%. Notably, the VAF increase from one to two synergies is greater than 0.05, whereas the increase from two to three is less than 0.05. Adding more synergies (three, four, or five) yields only marginal improvements, indicating a diminishing return. Therefore, two muscle synergies are sufficient to reconstruct over 90% of the original EMG signal across all movement conditions, and adding a third synergy does not materially improve reconstruction quality. As a result, the number of muscle synergies was fixed at two.

**FIGURE 2:**
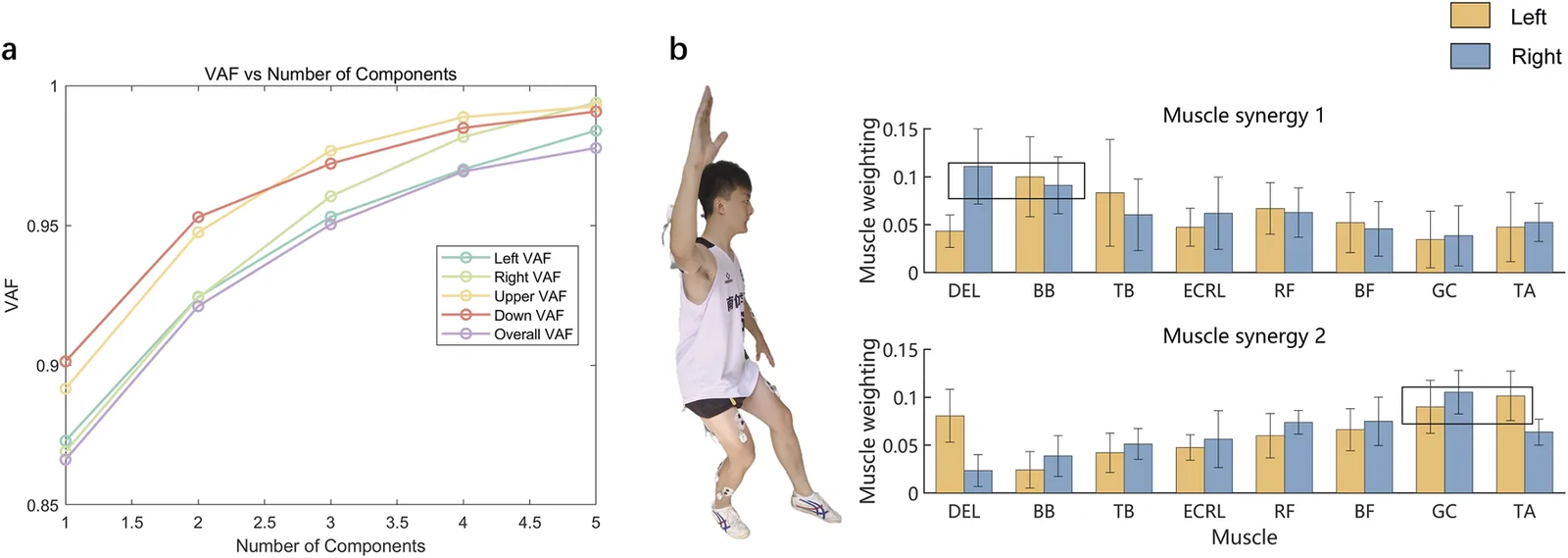
Muscle-synergy model order and a representative synergy pattern. **(a)** Relationship between the number of synergies and variance accounted for (VAF) for the complete 16-channel whole-body matrix and the independently decomposed left, right, upper-limb, and lower-limb submatrices. The smallest model order yielding VAF ≥0.90 was retained for each matrix; two synergies met this criterion for the whole-body matrix. **(b)** Muscle-synergy patterns during White Crane Spreads Its Wings. The final posture and the two whole-body synergy weightings are shown as mean ± standard deviation. Yellow and blue bars denote left- and right-side muscles, respectively. A weighting >0.1 is used descriptively to identify a prominent contribution and is not an inferential significance threshold. deltoid (DEL), biceps brachii (BB), triceps brachii (TB), extensor carpi radialis longus (ECRL), rectus femoris (RF), biceps femoris (BF), gastrocnemius (GC), and tibialis anterior (TA).

Across all 24 TC movements, the complete 16-channel whole-body matrices achieved a VAF of ≥0.90 with two synergies. Adding a third whole-body synergy increased VAF by < 0.05; therefore, the two-synergy solution was retained as the most parsimonious representation of whole-body muscle coordination. In the independently decomposed left-, right-, upper-limb-, and lower-limb submatrices, two synergies were insufficient to achieve a VAF of 0.90, whereas three synergies allowed every partition to meet this criterion. The three-synergy solutions reflect the greater complexity of muscle activation patterns within the corresponding anatomical partitions and were used only in the partition-specific analyses; they did not alter the model order of the independently derived whole-body decomposition.

### 3.2 Synergy patterns were repeatable across trials and participants

To evaluate the similarity of muscle synergies across subjects performing the same TC movement, cosine similarity was used as a metric. For each subject, the cosine similarity between the averaged synergy vector (computed from three repeated trials) and each individual trial was 0.9708 ± 0.0352, indicating that the average synergy vector reliably represents the synergy pattern for that specific subject and movement.

Furthermore, across all 23 participants and 24 TC movements, the average synergy vector for each movement was compared with the individual synergy vectors of all subjects. The resulting mean cosine similarity was 0.9281 ± 0.049, suggesting a high degree of consistency across individuals. This observation indicates that the 23 participants demonstrated stable and consistent muscle synergy patterns when performing the same TC movements. Therefore, the extracted synergy patterns for each movement can be considered representative of this sample of experienced practitioners.

### 3.3 Upper- and lower-limb modules dominated typical Tai Chi forms

The results of this study indicate that, despite differences in movement details, the 24 TC forms exhibit similar muscle synergy patterns. Through NNMF, two primary synergy vectors were consistently extracted across movements. One vector predominantly reflected strong activation in the upper limbs, while the other represented lower limb stabilization. This suggests that the human body relies on preconfigured, stable synergy modules rather than generating entirely new activation strategies for each movement.

As shown in Figure 2B, movement such as “White Crane Spreads Its Wings” demonstrate flexible combinations of these two synergy modules in terms of activation intensity and timing. This flexibility supports various demands for balance, force generation, and fine motor control.

In this TC movement, Synergy 1 (S1) and Synergy 2 (S2) are sequentially recruited to complete the action. S1 is primarily associated with right shoulder abduction. During this phase, the right elbow undergoes flexion, while the left elbow first extends and then flexes. This synergy is mainly composed of activations from the right deltoid, bilateral biceps brachii, and the left triceps brachii, reflecting a coordinated pattern of upper-limb muscle engagement. Synergy 2 (S2) is predominantly related to lower-limb activity and consists of the bilateral gastrocnemius and the left tibialis anterior, corresponding to plantarflexion and dorsiflexion at the ankle joint. During the movement, the right ankle remains relatively stable, whereas the left ankle transitions from dorsiflexion to plantarflexion.

These synergy patterns indicate that TC emphasizes concentrated utilization of the ankle joint while flexibly alternating between upper- and lower-limb activations, reflecting the movement’s integrative and balanced neuromuscular control strategy. The muscle synergy analysis results for other movements are provided in the Supplementary Material 2.

### 3.4 Bilateral muscle pairs showed the strongest coupling

Participant-level analysis supported stronger bilateral coupling. Mean ± SD NMI was 0.2682 ± 0.0299 for bilateral upper-limb connections, 0.2372 ± 0.0296 for ipsilateral upper-limb connections, 0.2688 ± 0.0352 for bilateral lower-limb connections, and 0.2335 ± 0.0351 for ipsilateral lower-limb connections. The bilateral-minus-ipsilateral difference was positive in all 23 participants (mean paired ΔNMI = 0.0332, 95% bootstrap CI [0.0322, 0.0342], exact two-sided p = 0.0020, Holm-adjusted p = 0.0059). There was no overall difference between upper- and lower-limb connections (ΔNMI = 0.0015, 95% CI [-0.0130, 0.0166], Holm-adjusted p = 0.8516). The interaction contrast was significant (ΔNMI = −0.0043, 95% CI [-0.0055, −0.0030], Holm-adjusted p = 0.0059), showing that the bilateral advantage was slightly greater for lower-limb than upper-limb connections.

The ranking analysis provided a similar descriptive pattern. Among the five highest-NMI pairs for each of the 24 movements (120 ranked pairs in total), 88.3% connected opposite sides of the body. Bilateral upper-limb pairs accounted for 43.3%, and bilateral lower-limb pairs accounted for 45.0%. These percentages are descriptive; the statistical conclusion is based on the participant-level analysis reported above.

Within-limb coupling was also observed, particularly between the right biceps brachii and right deltoid. However, cross-limb pairings across different limbs (e.g., left upper limb with right lower limb) showed no obvious patterns. These findings suggest that TC emphasizes strong bilateral inter-limb connectivity, underscoring its effectiveness in promoting symmetrical motor coordination. Detailed values for each movement are presented in Supplementary Material 3.

For “Left Grasping the Sparrow’s Tail,” the highest group-mean NMI was observed between BB_L and BB_R (mean NMI = 0.4687, 95% bootstrap CI [0.4221, 0.5232]). The next highest value was observed for DEL_R-BB_L (mean NMI = 0.4652, 95% CI [0.4154, 0.5225]). The label-permutation test identified 13 muscle pairs with NMI significantly higher than the randomized-label reference after BH-FDR correction (q ≤ 0.0485): DEL_R-BB_L, DEL_R-BB_R, DEL_R-TB_L, BB_L-BB_R, BB_L-TB_L, BB_R-TB_L, BB_R-TB_R, TB_L-TB_R, TB_R-ECRL_L, RF_R-BF_L, BF_R-GC_L, GC_L-GC_R, and GC_R-TA_L. These pairs are marked with asterisks in Figure 3. No pair had NMI significantly lower than the randomized-label reference after FDR correction. Thus, the marked pairs showed consistently strong relative coupling. However, the analysis did not provide evidence that any pair was physiologically separated.

**FIGURE 3:**
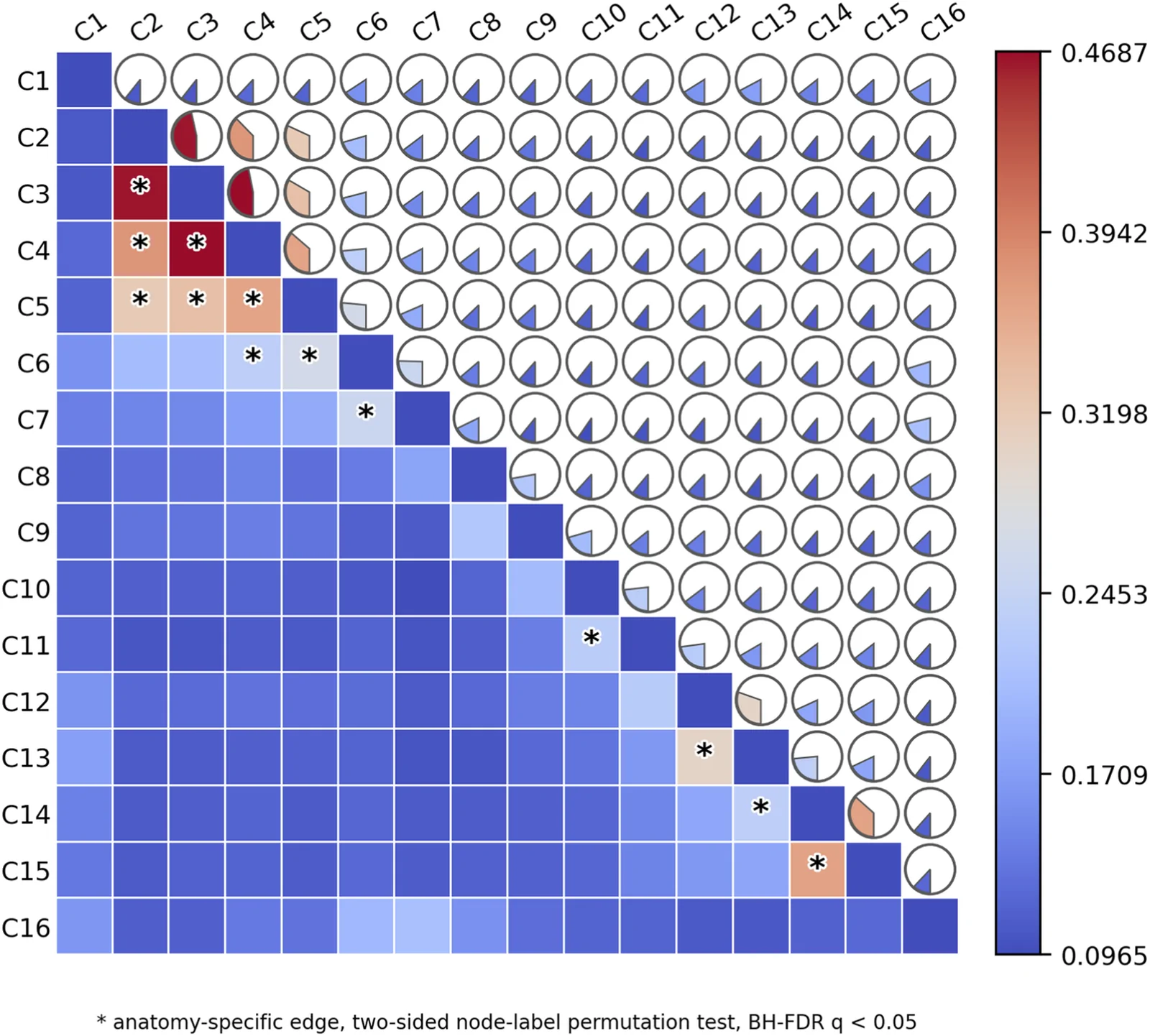
Descriptive group-averaged normalized mutual information (NMI) matrix for Left Grasping the Sparrow’s Tail. Warmer colors indicate stronger pairwise NMI. C1–C16 correspond to left deltoid, right deltoid, left biceps brachii, right biceps brachii, left triceps brachii, right triceps brachii, left extensor carpi radialis longus, right extensor carpi radialis longus, left rectus femoris, right rectus femoris, left biceps femoris, right biceps femoris, left gastrocnemius, right gastrocnemius, left tibialis anterior, and right tibialis anterior, respectively. The heat map is descriptive; inferential analyses are reported in the Results.

In other TC movements, some exhibit a chain-like connectivity among lower-limb muscles that spans the knee–ankle complex, reflecting a lower-limb-dominant coordination pattern. These movements also display pronounced coupling between the upper and lower limbs, with the two coupling patterns alternating and mutually reinforcing, producing extensive interlimb muscle coordination. NMI visualizations for the remaining movements are provided in the Supplementary Material 4.

### 3.5 Movement-specific networks exhibited small-world and modular organization

For each of the 24 movements, the group-level 16 × 16 NMI matrix was thresholded using the prespecified 0.01 scan and connectivity/degree rule. Each movement therefore had one group-level threshold selected from the same 0.00–1.00 candidate range; thresholds were not fitted separately for participants. Connections exceeding the selected movement-specific threshold were retained for the final functional muscle network. Figure 4a summarizes this workflow. The detailed results of the threshold calculations for each movement are presented graphically in Supplementary Material 5.

**FIGURE 4:**
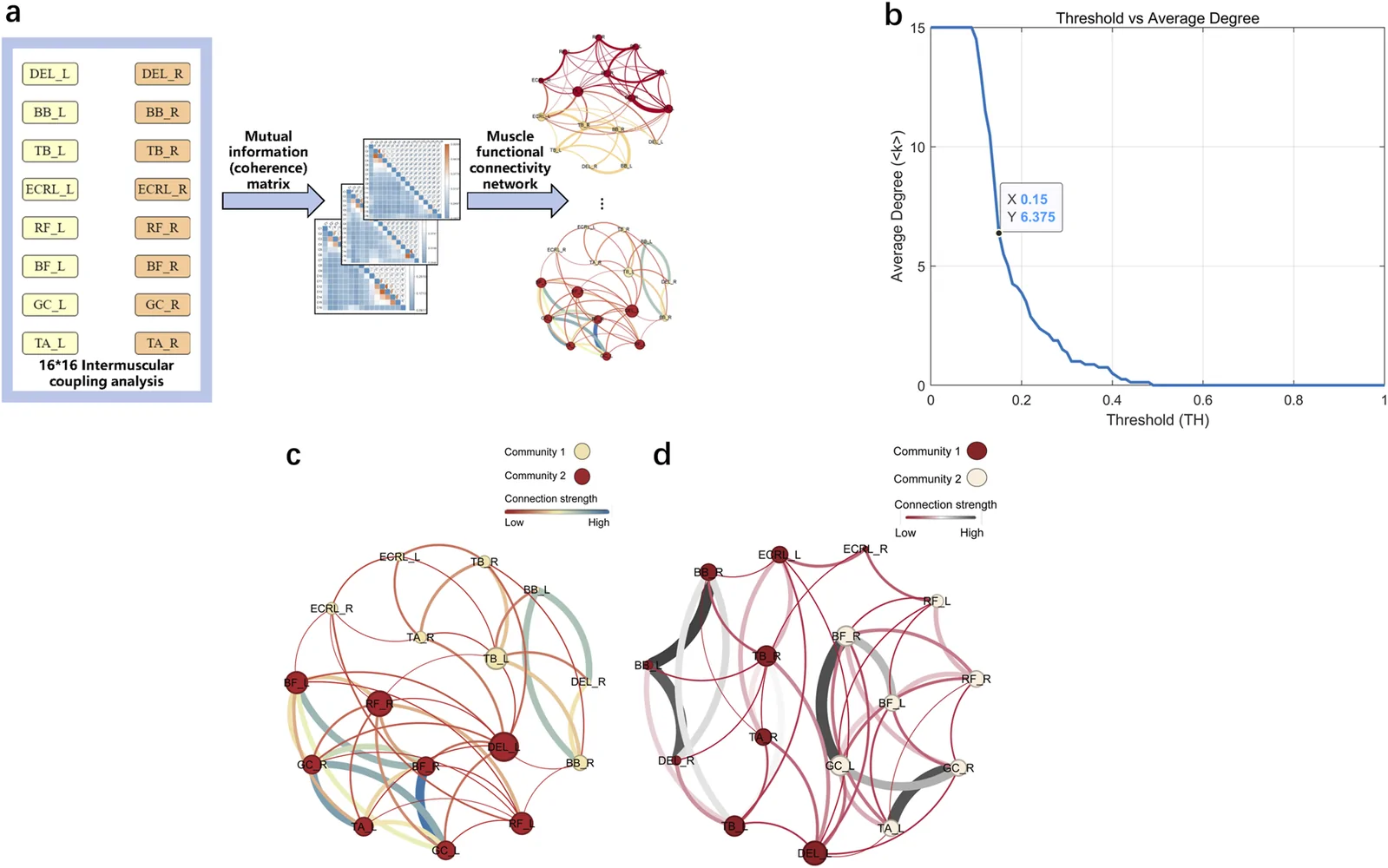
Construction and representative topology of the functional muscle network. **(a)** Workflow for constructing a movement-specific group network from participant-level NMI matrices averaged across three repetitions. Candidate thresholds from 0.00 to 1.00 were scanned in increments of 0.01, and the largest threshold retaining one connected component, no isolated nodes, and a mean degree >2ln (16) was selected. **(b)** Average node degree across candidate thresholds for Parting the Wild Horse’s Mane; T = 0.15 satisfied the prespecified connectivity criteria. **(c)** Functional network for Parting the Wild Horse’s Mane. **(d)** Functional network for Brush Knee and Twist Step. Larger nodes indicate higher degree centrality, thicker edges indicate higher NMI, and node colors denote communities with denser within-community than between-community coupling. Node labels use the left/right muscle abbreviations DEL_L/R, BB_L/R, TB_L/R, ECRL_L/R, RF_L/R, BF_L/R, GC_L/R, and TA_L/R. DEL: deltoid; BB: biceps brachii; TB: triceps brachii; ECRL: extensor carpi radialis longus; RF_R: rectus femoris (right); BF: biceps femoris; GC: gastrocnemius; TA: tibialis anterior. Yellow bars represent left-side muscles, and blue bars represent right-side muscles. If the muscle weightings exceed 0.1, it indicates that the muscle plays a significant role in contributing to the movement execution.

Using “Parting the Wild Horse’s Mane” as an example (Figure 4b), the candidate threshold of TH = 0.15 yielded an average node degree <k ≥ 6.375, which exceeds the theoretical threshold of 2ln (16) ≈ 5.5452, while maintaining full connectivity among all 16 muscle nodes. After binarization at this threshold, 51 out of 120 possible muscle pairs (42.5%) were retained as threshold-defined functional connections, forming a sparse network structure that highlights key patterns of intermuscular coordination.

Following successful construction of the muscle functional network, the muscle with the highest node degree was identified as the most engaged in the network. Additionally, muscles with high betweenness centrality were considered critical for coordination. Table 2 summarizes the key nodes (muscles), their corresponding node degrees, and betweenness centralities for all 24 TC forms, demonstrating variation in functional muscle network hubs across different movements.

**TABLE 2:** Key muscles of each movement and their corresponding node degree and betweenness centrality.

Index	Movement	Key muscle (Node)	Node degree	Node betweenness
1	Commencing form	TA-R	11	12.41
2	Parting the wild Horse’s mane	DEL-L	10	22.71
3	White crane spreads its wings	ECRL-R	13	21.17
4	Brush knee and twist step	DEL-L	8	43.44
5	Playing the lute	DEL-L	7	44.17
6	Repulse monkey	DEL-L	12	9.74
7	Grasp the Bird’s tail (left side)	BB-R	9	8.62
8	Grasp the Bird’s tail (right side)	DEL-L	10	34.79
9	Single whip 1	TA-R	10	13.83
10	Wave hands like clouds	DEL-L	13	31.25
11	Single whip 2	GC-L	13	20.50
12	High pat on horse	DEL-R	7	28.73
13	Right heel kick	BB-R	9	28.10
14	Strike to ears with both fists	RF-L	12	24.48
15	Turn and left heel kick	DEL-L	9	27.44
16	Left lower body and stand on one leg	BB-R	11	16.34
17	Right lower body and stand on one leg	DEL-L	8	36.35
18	Shuttle back and forth	RF-L, R	8	10.22
19	Needle at sea bottom	DEL-R	12	20.35
20	Fan through the back	RF-R	9	15.13
21	Turn, deflect, parry, and punch	DEL-L, ECRL-R	12	16.55
22	Apparent closing up	TB-L, TA-R	10	12.99
23	Cross hands	TA-R	12	28.88
24	Closing form	RF-L, R	8	18.47

TA, tibialis anterior; DEL, deltoid; BF, biceps femoris; TB, triceps brachii; ECRL, extensor carpi radialis longus; GC, gastrocnemius; BB, biceps brachii; RF, rectus femoris; L, left; R, right.

Modularity analysis classified the 24 TC forms into two categories: one group formed two communities (e.g., Parting the Wild Horse’s Mane), while the other formed three communities (e.g., Repulse Monkey). This suggests that muscle groups exhibit both strong global coordination and localized clustering, characterized by densely connected sub-networks and relatively sparse inter-module connections, revealing a complex hierarchical structure of intermuscular coordination.

Further network visualization and topological analysis revealed that in Parting the Wild Horse’s Mane, the left deltoid (DEL_L) emerged as the primary functional hub with the highest node degree (k = 10), indicating a dominant role during movement execution. Additionally, the lower and upper limb muscles formed distinct functional communities, with the left deltoid serving as an intermodular hub that bridges these communities (Figure 4c).

In another representative movement, “Wave Hands Like Clouds”, the left deltoid reaches a node degree of 12. Modular analysis divides the muscle system into three functional communities corresponding to lower-limb stabilization, dynamic balance, and upper-limb motion. This structure highlights the necessity for tighter inter-muscle coordination in executing more complex movements. Schematic diagrams of the muscle functional connectivity networks for the other movements are provided in the Supplementary Material 6.

Additionally, quantitative analysis of network topology across all 24 TC movements reveals a consistent small-world architecture, characterized by high clustering coefficients (mean γ = 1.72 ± 0.22), short average path lengths (λ ≈ 1), and small-worldness indices (σ = 1.77 ± 0.25). These properties indicate an efficient balance between local specialization and global integration. The average path length of only 2.93 ± 0.88 suggests that functional coordination between any two muscle groups can be achieved within just two to three steps. Notably, during the closing phase of movements, increased local connectivity and shorter transmission paths further support the neuromuscular efficiency inherent to TC practice.

Statistical analysis revealed that the modularity index Q for the majority of movements ranged between 0.23 and 0.41, with 62.5% of the movements exhibiting Q values exceeding the commonly accepted resolution threshold of 0.3 (as per Newman’s criterion (Newman, 2006)). The remaining nine movements (37.5%) had Q ≤ 0.30 and were interpreted as showing weaker or less clearly separated communities rather than meaningful modular organization. Table 3 presents descriptive network metrics.

**TABLE 3:** Network analysis parameters and small-world characteristics of each movement.

Index	Movement	Modularity	C	L	γ	λ	σ
1	Commencing form	0.256	0.51	2.01	1.50	0.97	1.54
2	Parting the wild Horse’s mane	0.333	0.36	3.40	1.81	0.99	1.82
3	White crane spreads its wings	0.288	0.42	2.81	1.51	0.99	1.52
4	Brush knee and twist step	0.414	0.32	3.64	2.03	0.96	2.12
5	Playing the lute	0.296	0.43	2.44	1.62	0.97	1.67
6	Repulse monkey	0.293	0.25	5.46	1.38	1.01	1.37
7	Grasp the Bird’s tail (left side)	0.356	0.30	4.24	1.66	1.00	1.67
8	Grasp the Bird’s tail (right side)	0.385	0.32	4.03	1.77	0.98	1.80
9	Single whip 1	0.333	0.38	2.89	1.71	0.97	1.76
10	Wave hands like clouds	0.326	0.31	4.11	1.63	1.01	1.61
11	Single whip 2	0.288	0.43	2.69	1.41	1.00	1.41
12	High pat on horse	0.358	0.43	2.30	1.79	0.97	1.86
13	Right heel kick	0.254	0.39	2.38	1.59	0.98	1.63
14	Strike to ears with both fists	0.228	0.44	2.28	1.85	1.00	1.84
15	Turn and left heel kick	0.351	0.46	2.20	2.02	0.97	2.08
16	Left lower body and stand on one leg	0.320	0.35	3.17	1.58	0.99	1.60
17	Right lower body and stand on one leg	0.396	0.40	2.68	1.87	0.97	1.94
18	Shuttle back and forth	0.395	0.35	3.11	1.94	0.94	2.06
19	Needle at sea bottom	0.284	0.44	2.47	1.62	0.97	1.67
20	Fan through the back	0.355	0.51	2.09	2.12	0.94	2.25
21	Turn, deflect, parry, and punch	0.315	0.39	3.53	1.56	0.98	1.60
22	Apparent closing up	0.247	0.36	2.30	1.52	0.93	1.62
23	Cross hands	0.334	0.50	2.45	1.60	0.97	1.65
24	Closing form	0.406	0.57	1.78	2.15	0.93	2.32

C , Average clustering coefficient; L , Average path length; γ , Clustering coefficient ratio; λ , Path length ratio; σ , Small-world coefficient.

Analysis of specific movements revealed that “Brush Knee and Twist Step” exhibited the highest level of modularity, with a Q value of 0.41. This suggests that the neuromuscular control system may adopt a hierarchical division of labor strategy to reduce the demand for cross-module information exchange, reflecting an efficient internal organization and functional specialization of the motor control system. A highly modular network structure indicates the presence of well-separated functional units. Within this structure, intra-module connections are dense, while inter-module connections remain sparse. Although such a modular configuration enhances the precision of motor control, it may also increase the risk of movement failure during rehabilitation, as patients may have limited capacity for cross-module reorganization. A schematic representation of the muscle functional connectivity network for this movement is shown in Figure 4d.

## 4 Discussion and conclusion

To analyze the biomechanical coordination patterns and interrelationships among limb-related muscle groups during TC practice, this study employed mutual information-based quantification of muscle functional networks. This approach offers valuable insights into the neuromuscular coordination mechanisms involved in different TC movements and across various body segments. By further analyzing muscle synergies, the study identifies key contributing muscles for each movement. These findings provide a biomechanical basis for understanding the neuromuscular coordination characteristics of Tai Chi, which may inform future research into its potential applications in neurological rehabilitation.

### 4.1 Robustness of synergy extraction method

The consistency in the number of optimal muscle synergies and the high similarity of weight vectors across different sample conditions (cosine similarity index r ranging from 0.71 to 0.98) (Akoglu, 2018) indicate the robustness of the synergy extraction method. The extracted synergies generally show dominant contributions either from upper limb muscles such as the deltoid and biceps brachii, or from lower limb muscles like the gastrocnemius and tibialis anterior. Taking the “White Crane Spreads Its Wings” movement as an example, two muscle synergies were extracted, accounting for 90% of the movement variance. Synergy two primarily involved GC-R and TA-L, which were activated to facilitate ankle dorsiflexion and plantarflexion. Muscle synergy analysis and other biomechanical assessments of lower limb movements during Tai Chi have also demonstrated significant activation of ankle-related muscles. This is evidenced by increased peak EMG and RMS EMG amplitudes (Wu et al., 2004), suggesting that fine adjustments at the ankle joint ensure effective torque transmission (Zhang et al., 2024). The potential mechanism of TC training may be associated with shortened activation delays in lower limb muscles, particularly the tibialis anterior and gastrocnemius (Sun et al., 2016; Wang et al., 2017). Given the tibialis anterior’s crucial role in maintaining postural stability (Huang et al., 2024), its activation is especially significant. Recent analysis of elite TC heel-kick performance likewise found largely conserved bilateral synergy composition with leg-specific temporal modulation, supporting the distinction between stable module structure and task-dependent recruitment timing (Mo and Jing, 2025).

### 4.2 Dominant synergy contributions

Notably, in some movements, such as “Single Whip,” the extracted synergies showed high contributions from the biceps femoris and rectus femoris. For example, synergy two was dominated by rectus femoris activation, likely due to the knee-lifting action involved. This motion effectively engages muscles around the knee, enhancing the strength and stability of the knee flexor-extensor groups (Li H. et al., 2023). Synergy 1, on the other hand, was characterized by activation of DEL-L, BB-L, and BB-R, contributing to shoulder abduction and elbow flexion. Furthermore, a corticomuscular coupling study found that during TC practice, patients exhibited increased coherence between the biceps brachii and contralateral cortical regions. This suggests a compensatory mechanism via enhanced muscle activation to offset reduced corticomuscular coupling, highlighting TC’s potential in promoting cortical-muscular integration (Zu et al., 2023).

### 4.3 Intermuscular coupling characteristics of Tai Chi

Existing literature has yet to provide detailed insights into the dynamic coupling between muscles during TC movements. Most prior studies have focused on absolute changes in muscle force during TC, without considering inter-muscular relationships (Wu et al., 2004; Huang et al., 2024; Li H. et al., 2023). In contrast, our findings reveal that TA-L and GC-R frequently exhibit strong functional connectivity across a variety of movements. Furthermore, most muscle pairs with high NMI values are composed of left- and right-side muscle combinations, supporting the bilateral nature of TC movements (Li et al., 2012; Yu et al., 2020).

This pattern of repeated force transfer between sides is particularly relevant for addressing post-stroke asymmetrical posture, which is a common motor impairment in stroke survivors (Kim et al., 2015). In addition, efficient activation of the tibialis anterior has been linked to improved anterior-posterior center of mass movement, which plays a critical role in enhancing postural stability and balance control (Opattova et al., 2015). TC also emphasizes broad connectivity among upper limb muscles, which may help explain its effectiveness in restoring upper limb function in stroke rehabilitation (Zhao et al., 2022). Notably, our results also demonstrate that some movements exhibit extensive coupling among lower limb muscles, a pattern that is foundational for efficient weight shifting and dynamic postural control during TC (Zhao et al., 2021).

According to the motor control mechanisms of the central nervous system (CNS), stroke patients exhibit altered muscle activation strategies during movement compared to healthy individuals due to brain injury. These changes often result in compensatory muscle responses and abnormal patterns of muscle coordination. In upper limb movements, for example, stroke patients tend to rely excessively on the biceps brachii to perform motor tasks, owing to impaired function of the deltoid and triceps brachii, leading to a phenomenon known as abnormal muscle synergy (Sheng et al., 2024). Muscle activation on the non-paretic side typically precedes that on the paretic side, and activation on the paretic side tends to occur in a distal-to-proximal sequence (Prudente et al., 2013). Furthermore, impaired coordination on the paretic side forces the non-paretic leg to reduce neuromuscular complexity in order to compensate for deficits during sit-to-stand transitions (Li YA. et al., 2023). On the other hand, poor temporal coupling between the knee flexors and extensors after stroke contributes to difficulties during postural transitions (Kerr et al., 2019).

### 4.4 Neural and neurophysiological substrates of Tai Chi

Using sEMG across the 24-form Tai Chi, we observed integrated, coordinated muscle activation rather than isolated contractions—a pattern that likely underlies Tai Chi’s smoothness and stability. Previous studies have shown that TC practice significantly activates the supplementary motor area (SMA) of the brain—a key region responsible for motor sequence planning. By regulating subcortical structures, the SMA facilitates the temporal integration of upper and lower limb coordination modules, highlighting the complexity of motor orchestration involved in TC (Chen XP. et al., 2021). Moreover, Tai Chi practice has been shown to induce cortical synchronization of alpha oscillations and desynchronization of slow delta oscillations (He and Hu, 2022), The former facilitates enhanced motor preparation and attentional regulation, while the latter reduces background neural noise, thereby providing a neurophysiological basis for fine motor control. Further studies on cortico-muscular coupling have revealed that, although the direct coupling strength between the cortex and muscles is reduced, muscle activity is enhanced. This suggests that subcortical pathways—such as the reticulospinal and rubrospinal tracts—may compensate for diminished cortical control, enabling multi-level and multi-pathway coordination (Zu et al., 2023; Conway et al., 1995). Collectively, these findings support the notion that Tai Chi engages unique neuromotor regulatory mechanisms, providing a theoretical foundation for its application in enhancing motor performance and promoting rehabilitation. A health-neuroscience perspective frames such effects as reciprocal brain-body adaptations arising from sustained behavior and motor practice (Tang and Tang, 2024). Consistent with this interpretation, Tai Chi practitioners have shown altered lower-limb corticomuscular coherence during balance-demanding conditions, although those observations do not establish causality for the present muscle-network findings (Hu et al., 2025).

### 4.5 Clinical implications

These synergy- and mutual-information–derived findings can directly inform precision training protocols. Prioritize network hubs and high-degree nodes (e.g., the ankle complex and key shoulder/knee muscles) as primary targets for strengthening or neuromuscular retraining. Select and sequence movements according to their dominant synergy type—upper-limb, lower-limb chain, knee-dominant, interlimb/cross-limb, balance/weight-shift, or complex whole-body sequences—to meet specific functional goals. Use EMG, NMI, and modularity metrics to monitor intervention effects and individualize progression. Practically, upper-limb–dominant movements support shoulder–elbow coordination and fine motor control; lower-limb chain movements train weight transfer, push-off/landing mechanics, and gait; knee-dominant movements target quadriceps/hamstring strength and knee stability; interlimb coupling movements restore bilateral symmetry and contralateral coordination; ankle-hub balance/weight-shift movements improve postural control and fall-prevention; and complex whole-body sequences develop motor planning, timing, and multi-module integration (e.g., advanced sequencing or dual-task training).

The participants in this study were healthy, experienced TC practitioners. Therefore, the identified muscle synergy structures and patterns of functional connectivity represent normative neuromuscular coordination rather than pathology- or recovery-related adaptations. Extrapolation of these findings to stroke patients or other clinical populations should be undertaken with caution and requires further empirical validation.

### 4.6 Limitations

This pilot study has several limitations. First, the sample comprised only 23 healthy, experienced practitioners and did not include a novice, non-Tai Chi, or clinical control group; the findings therefore describe this specialist sample and should not be generalized to patients. Second, training duration was recorded only in broad categories, and weekly practice frequency was not collected, preventing dose-response or subgroup analysis and leaving experience variability as a potential confounder. Finally, only limb muscles were recorded; trunk muscles, which contribute substantially to postural control, were not assessed. Larger prospective studies should collect continuous training-dose data, standardize rest and familiarization, include appropriate control groups and trunk sEMG, and prespecify confirmatory statistical analyses.

## 5 Conclusion

This pilot study shows that multi-channel sEMG and muscle-synergy analysis can describe coordinated muscle activity during Tai Chi. The two-synergy whole-body model achieved VAF >90% and showed high consistency across trials (0.9708 ± 0.0352) and participants (0.9281 ± 0.049). In the 23-participant NMI subset, bilateral connections were stronger than ipsilateral connections within the same limb region (mean paired ΔNMI = 0.0332, Holm-adjusted p = 0.0059). This suggests that optimizing motor control efficiency may be achieved through enhanced coupling and reconstruction of contralateral multi-joint coordination. The presence of cross-limb mirrored synergies further implies potential therapeutic value in restoring functional impairments via reinforcement of these coupling mechanisms. Furthermore, the muscle functional network displays hierarchical regulation and a small-world topology (σ > 1), with the ankle muscle complex (gastrocnemius–tibialis anterior) serving as a central hub in balance control, providing a key biomechanical target for optimizing fall-prevention strategies in Tai Chi training.

## Data Availability

The original contributions presented in the study are included in the article/Supplementary Material, further inquiries can be directed to the corresponding authors.
