# Supplementary material for: Muscle synergies and functional network organization underlying Tai Chi movements: a pilot cross-Sectional study towards a personalized Neuromechanical Baseline
Source: Front Bioeng Biotechnol. 2026 Sep 18;14:1928241. doi: 10.3389/fbioe.2026.1928241 (PMC13631746; doi:10.3389/fbioe.2026.1928241)
Supplement: Supplementary file 1 [file Supplementaryfile1.docx]

**Supplementary Material 1.** The supplementary method.

a. Muscle Synergy Extraction

- Non-Negative Matrix Factorization (NNMF)

Based on the preprocessed and normalized sEMG envelopes, NNMF was applied to extract muscle synergies[1]. NNMF decomposes the original sEMG envelope matrix into two non-negative matrices: a basis matrix $W$, representing the weighting of each muscle's contribution to specific synergies, and a coefficient matrix $H$, representing the time-varying activation patterns of these synergie[2]. The decomposition can be expressed as:

$V\approx W\cdot H$

Here,$V$is an $m\times n\text{ }$ matrix (where$m$ is the number of muscle channels, and$n$ is the number of time samples).$W$ represents the characteristic patterns of each muscle, structured as$m\times r\text{ }$(where $r$ is the number of extracted synergies), $H$ represents the activation level of each synergy over time, structured as $r\times n$. The activation coefficients$H$ describe the temporal modulation of sEMG (the temporal component of motor control), while the weight vectors $W$describe the spatial contributions of the synergies (the spatial component of motor control)[3]. $W$and$H$ are updated iteratively to minimize the difference between $W\cdot H$and $V\text{ }$, with the error recorded after each iteration. Results are returned once the maximum iteration count is reached or convergence occurs.

The reconstruction accuracy of the original sEMG matrix by the decomposed model is expressed through Variance Accounted For (VAF), which describes the proportion of total variance in the original data explained by the decomposition model. The VAF value ranges between 0 and 1, where a larger value indicates higher reconstruction quality. It is calculated using the following formula:

$$\text{VAF}=1-\frac{\text{‖}V-WH\text{‖}_{F}^{2}}{\text{‖}V\text{‖}_{F}^{2}}$$

The Frobenius norm representing the square root of the sum of the squares of all elements in a matrix, was employed in this study to determine the optimal number of muscle synergies(k) required to accurately reconstruct the original sEMG matrix $V$. The selection of k was guided by ensuring a Variance Accounted For (VAF) of ≥90% (a global standard)[4, 5]. After determining k for each participant in every training session, the common number of muscle synergies was identified as the synergy number most consistently observed across individuals within the group[6]. The 16-channel sEMG signals were further divided into submatrices based on laterality (left and right) and limb segmentation (upper and lower limbs) to ensure that the VAF for each submatrix also met or exceeded 90%. If the submatrix VAF was ≥90%, it was concluded that no additional synergies were required to reconstruct the original sEMG envelope. Conversely, if VAF <90%, the number of muscle synergies was incrementally increased until all submatrix VAF values reached or exceeded 90%[3].

Since the order of muscle synergies extracted by NNMF varies randomly across subjects and repetitions[6], cosine similarity was computed to reorder the synergy vectors prior to averaging, allowing for the calculation of a representative synergy pattern. Cosine similarity quantifies the similarity between two vectors by normalizing their dot product with the product of their magnitudes[7]. The formula is as follows:

$$\text{Cosine Similarity}=\frac{\mathbf{A}\cdot\mathbf{B}}{\text{‖}\mathbf{A}\text{‖‖}\mathbf{B}\text{‖}}$$

For two vectors$\mathbf{A}$ and $\mathbf{B}$, $\mathbf{A}\cdot\mathbf{B}$ represents their dot product, while $\text{‖}\mathbf{A}\text{‖}$ and $\text{‖}\mathbf{B}\text{‖}$ denote the norms of the respective vectors. Cosine similarity values close to 0 and 1 indicate dissimilarity and high similarity, respectively. Based on similar studies, a cosine similarity value exceeding 0.684 is considered indicative of similarity[8].

First, the three repeated trials of each subject were aligned and averaged based on cosine similarity to obtain individual representative muscle synergy patterns. Then, for a selected reference participant, the cosine similarity between each synergy vector and the corresponding vectors from all other participants was calculated[9]. Synergies with high similarity were grouped to create an average muscle weighting set. Finally, to verify whether the resulting average synergy patterns could represent the motor behavior of all participants, the cosine similarity between each vector of the average synergy pattern and the synergy vectors of each individual participant was computed.

b. Computation of Normalized Mutual Information

Normalized Mutual Information(NMI)

To quantify the interactions between muscle groups, we computed the NMI between muscle pairs. NMI measures the amount of shared information between two variables or time series signals[10] and is widely used as an index of **functional connectivity** to highlight dynamic coupling between muscles[11]. The first step involves calculating the average information contained in the sEMG signal$X$, defined as entropy$H$, The formula for information entropy is expressed as follows:

Here, or represents the probability of the random variable$X$ or$Y$ falling into the $i$- th bin, estimated using the histogram method. The term $\log$ denotes the natural logarithm, and is a small positive value introduced to prevent numerical issues when computing$log(0)$. Higher values of $H(X)$ or $H(Y)$ indicate a more uniform signal distribution and greater uncertainty. In mutual information calculations, entropy serves as a critical normalization benchmark. The formula for mutual information between channel $X$ and channel $Y$ is given as follows:

$$I(X;Y)=\sum_{i} \sum_{j} P_{XY}(i,j)\log\frac{P_{XY}(i,j)}{P_{X}(i)P_{Y}(j)}$$

Here, represents the joint probability distribution of $X$ and $Y$ while and denote the marginal probability distributions of $X$ and $Y$ , respectively. Mutual Information (MI) is a non-negative quantity; it equals 0 when $X$ and $Y$ are completely independent, and its upper bound is the minimum entropy of $X$ and $Y$ , which occurs when $X$ and $Y$ are fully correlated (i.e., when MI equals 1). To establish a common, measurable value for different participants for ease of comparison, the **NMI** is calculated using the following formula:

$$\text{NMI}(X,Y)=\frac{I(X;Y)}{\min(H(X),H(Y))}$$

Here, $min(H(X),H(Y))$ represents the smaller value between $H(X)$ and $H(Y)$ , which is used to define the range for the NMI, with values between [0, 1]. Here, the smaller marginal entropy is used for normalization, so NMI ranges from 0 to 1. Sixteen muscles produce 120 unique undirected muscle pairs after diagonal self-connections are excluded. For each participant and movement, the NMI matrix was obtained by averaging the three repetitions. The group matrix was then calculated by averaging the matrices of the 23 participants. For the additional statistical analysis, the participant was treated as the independent analysis unit. Each participant's NMI values were averaged across all 24 movements within four predefined groups: bilateral upper-limb, ipsilateral upper-limb, bilateral lower-limb, and ipsilateral lower-limb connections. Three exact two-sided sign-flip tests compared bilateral with ipsilateral connections, upper- with lower-limb connections, and the interaction between these two factors. All 2^10 sign patterns were tested. Holm correction was applied to the three comparisons, and 95% confidence intervals were calculated using 50,000 participant-level bootstrap samples. The 120 muscle pairs were tested using 100,000 within-participant muscle-label permutations. Two-sided p values were corrected using BH-FDR. These tests determine whether a muscle pair has higher or lower NMI than expected after the anatomical labels are randomized. Because only repetition-averaged NMI matrices were available, the tests cannot establish absolute independence of the original sEMG signals. Low or non-significant NMI was therefore not interpreted as physiological separation.

c. Functional Network Construction and Characteristics Extraction

Based on the NMI matrix (representing the correlation features between signals), an $n\times n$ adjacency matrix is constructed. The rows and columns represent the nodes, and represents the NMI between channel $i$ and channel $j$. By selecting an appropriate threshold $TH$, the matrix is simplified into a binary undirected network. If the NMI is greater than or equal to $TH$, then is set to 1, establishing an edge between channel $i$ and channel $j$; otherwise, no edge is established[12].

The threshold selection range should ensure the completeness of the network, meaning the network should not contain isolated nodes or disconnected components. It should also guarantee the small-world property of the network, which implies a high clustering coefficient, a small average path length, and an average degree greater than $2Inn$, ensuring the network has low global efficiency and high local efficiency[12, 13].

Analyzing the node characteristics of the muscle functional network[14] helps to understand the differences between networks in different movements and plays an important role in identifying the connections between key muscle nodes. Therefore, we evaluate the effect of different thresholds on network properties by calculating the average degree($\langle k\rangle$)of the mutual information matrix. First, based on sparse network theory, which ensures the basic connectivity of the network structure, the minimum expected value of the average degree is defined as follows:

$$\langle k\rangle_{\text{min}}=2\log(n)$$

Here, $n$ represents the dimension of the mutual information matrix, i.e., the number of network nodes. Then, based on each$\langle k\rangle$, a binary adjacency matrix is constructed. For each node $i$ its degree()is defined as:

$$k_{i}=\sum_{j} A_{ij}$$

The larger the degree of a node, the more important the node is in the network. The average degree of all nodes is calculated and recorded as the average degree of the entire network:

$$\langle k\rangle=\frac{1}{n}\sum_{i=1}^{n} k_{i}$$

**Analyzing the nodal characteristics** of the muscle functional network[14] helps reveal differences across movement patterns and plays a crucial role in identifying the connections between key muscle nodes. In particular, we calculated the **betweenness centrality** of each node in the network. For a given node $v$ , its betweenness centrality is defined as:

$$C_{B}(v)=\sum_{s\neq v\neq t} \frac{\sigma_{st}(v)}{\sigma_{st}}$$

Here, $C_{B}(v)$ represents the betweenness centrality of node $v$, $\sigma_{st}(v)$ denotes the number of shortest paths passing through node $v$, and $\sigma_{st}$indicates the total number of shortest paths between nodes $s$ and $t$. A higher betweenness centrality implies that the node serves as a critical bridge within the network, helping to identify muscles that play a key role in movement coordination. The degree helps recognize muscles with higher participation. In this study, muscles with both high degree and high betweenness centrality were identified as key muscles for the specific movement.

This study will compute key graph-theoretical metrics of the muscle functional network—including modularity, characteristic path length, average clustering coefficient, and small-worldness—to explore network differences across various Tai Chi movements. These metrics can be broadly categorized into integration and segregation indices based on their functional implications. Specifically, characteristic path length and small-worldness reflect the network’s capacity for global integration of information, while average clustering coefficient and modularity characterize the degree of local specialization or functional segregation within the network[15, 16].

Meanwhile, after constructing the network based on the threshold, we analyzed two graph theory network parameters, clustering coefficient (representing segregation) and average shortest path length (representing integration), to estimate the global structural differences of the functional network[15]. The clustering coefficient of node $i$() reflects the ratio of actual connections among its neighboring nodes to the theoretical maximum connections possible. It is defined by the following formula:

$C_{i}=\frac{2E}{k_{i}(k_{i}-1)}$

Here, $E$ is the number of actual connections among the neighboring nodes of node $i$; and $k_{i}$ is the degree of node $i$(i.e., the number of its neighboring nodes). The average clustering coefficient of the network($C$)is the mean of the clustering coefficients of all nodes:

$$C=\frac{1}{n}\sum_{i=1}^{n} C_{i}$$

Here, $n$ represents the total number of nodes in the network. A higher clustering coefficient indicates greater local clustering within the network, which is typically observed in complex networks such as social or biological networks, reflecting their tight connectivity properties. The higher the clustering coefficient, the better the network connectivity. Similarly, a larger average degree implies stronger correlations between channels, highlighting the network topology’s role in promoting cooperative behaviors[12].

Meanwhile, path length represents the average distance between any two nodes. The global average path length, used to measure the integration of the network, is calculated by averaging the characteristic path lengths of all nodes 𝑖∈𝑛 in the network[15]. It is defined by the following formula:

$$L=\frac{1}{n(n-1)}\sum_{i\neq j} \frac{1}{D(i,j)}$$

Here $D(i,j)$ represents the shortest path length between node 𝑖 and node 𝑗, and the diagonal elements $D(i,j)$= 0 are excluded from the calculation.

To demonstrate whether the muscle functional networks exhibit small-world properties, which are defined by a high clustering coefficient compared to a random network and relatively short path length[15], the small-world property metrics were calculated as follows:

$\sigma=\frac{\gamma}{\lambda}=\frac{C/C_{\text{rand}}}{L/L_{\text{rand}}}$

Here, $Crand$ represents the average clustering coefficient of the corresponding random network, and $Lrand$ represents the average path length of the corresponding random network. A network is considered to exhibit small-world properties only if the following conditions are simultaneously satisfied: γ > 1, λ ≈ 1, σ > 1.

Modularity is a quality metric for network partitioning. Its core concept is to divide a network into modules (or communities) such that nodes within the same module are more densely connected, while connections between different modules are sparser[17, 18] . In this study, modularity analysis is applied to the sEMG network to identify functional modules. This approach helps us better understand the coordination and division of labor among different muscle groups, shedding light on the underlying mechanisms of motor control. In this study, the resolution parameter was set to the standard value of 1.0. To enhance the stability of the results, nodes were processed in a randomized order, and weighted networks were employed for all analyses. The definition of modularity is as follows:

$$Q=\frac{1}{2m}\sum_{i,j} \left(A_{ij}-\frac{k_{i}k_{j}}{2m}\right)\delta(c_{i},c_{j})$$

Where $m$ represents the total number of edges in the network, denotes the edge weight between nodes $i$ and$j$, and $\delta(c_{i},c_{j})$ equals 1 if nodes $i$ and$j$belong to the same community, otherwise 0. Additionally, $k_{i}$and represent the degrees of nodes $i$ and$j$, respectively. This formula represents the difference between the actual connections of nodes and the expected connections in a random network. A higher modularity value indicates a more pronounced community structure, meaning that connections within the same community are significantly more frequent than would occur by chance. Generally, a modularity value greater than 0.3 is considered indicative of a significant and well-defined community structure.

[1]	D. D. Lee, and H. S. Seung, “Learning the parts of objects by non-negative matrix factorization,” *Nature,* vol. 401, no. 6755, pp. 788-91, Oct 21, 1999.

[2]	J. Taborri, V. Agostini, P. K. Artemiadis, M. Ghislieri, D. A. Jacobs, J. Roh, and S. Rossi, “Feasibility of Muscle Synergy Outcomes in Clinics, Robotics, and Sports: A Systematic Review,” *Appl Bionics Biomech,* vol. 2018, pp. 3934698, 2018.

[3]	M. Ghislieri, M. Knaflitz, L. Labanca, G. Barone, L. Bragonzoni, M. G. Benedetti, and V. Agostini, “Muscle Synergy Assessment During Single-Leg Stance,” *IEEE Trans Neural Syst Rehabil Eng,* vol. 28, no. 12, pp. 2914-2922, Dec, 2020.

[4]	D. J. Clark, L. H. Ting, F. E. Zajac, R. R. Neptune, and S. A. Kautz, “Merging of healthy motor modules predicts reduced locomotor performance and muscle coordination complexity post-stroke,” *J Neurophysiol,* vol. 103, no. 2, pp. 844-57, Feb, 2010.

[5]	V. R. Barradas, J. J. Kutch, T. Kawase, Y. Koike, and N. Schweighofer, “When 90% of the variance is not enough: residual EMG from muscle synergy extraction influences task performance,” *J Neurophysiol,* vol. 123, no. 6, pp. 2180-2190, Jun 1, 2020.

[6]	Y. A. Li, Z. J. Chen, C. He, X. P. Wei, N. Xia, M. H. Gu, C. H. Xiong, Q. Zhang, T. M. Kesar, X. L. Huang, and J. Xu, “Exoskeleton-Assisted Sit-to-Stand Training Improves Lower-Limb Function Through Modifications of Muscle Synergies in Subacute Stroke Survivors,” *IEEE Trans Neural Syst Rehabil Eng,* vol. 31, pp. 3095-3105, 2023.

[7]	A. S. Oliveira, L. Gizzi, D. Farina, and U. G. Kersting, “Motor modules of human locomotion: influence of EMG averaging, concatenation, and number of step cycles,” *Front Hum Neurosci,* vol. 8, pp. 335, 2014.

[8]	A. Oshima, Y. Nakamura, and K. Kamibayashi, “Modulation of Muscle Synergies in Lower-Limb Muscles Associated With Split-Belt Locomotor Adaptation,” *Front Hum Neurosci,* vol. 16, pp. 852530, 2022.

[9]	B. Kibushi, S. Hagio, T. Moritani, and M. Kouzaki, “Speed-Dependent Modulation of Muscle Activity Based on Muscle Synergies during Treadmill Walking,” *Front Hum Neurosci,* vol. 12, pp. 4, 2018.

[10]	I. Junquera-Godoy, J. L. Martinez-De-Juan, G. González-Lorente, J. M. Carot-Sierra, J. Gomis-Tena, J. Saiz, S. García-Blasco, I. Pertusa-Mazón, E. Soler-Climent, and G. Prats-Boluda, “Muscle Network Connectivity Study in Diabetic Peripheral Neuropathy Patients,” *Sensors (Basel),* vol. 24, no. 15, Jul 31, 2024.

[11]	P. Madeleine, Y. Xie, G. P. Y. Szeto, and A. Samani, “Effects of chronic neck-shoulder pain on normalized mutual information analysis of surface electromyography during functional tasks,” *Clin Neurophysiol,* vol. 127, no. 9, pp. 3110-3117, Sep, 2016.

[12]	L. Chen, X. Liu, B. Xuan, J. Zhang, Z. Liu, and Y. Zhang, “Selection of EMG Sensors Based on Motion Coordinated Analysis,” *Sensors (Basel),* vol. 21, no. 4, Feb 6, 2021.

[13]	R. I. Goldman, J. M. Stern, J. Engel, Jr., and M. S. Cohen, “Simultaneous EEG and fMRI of the alpha rhythm,” *Neuroreport,* vol. 13, no. 18, pp. 2487-92, Dec 20, 2002.

[14]	J. Wang, C. Yang, R. Wang, H. Yu, Y. Cao, and J. Liu, “Functional brain networks in Alzheimer’s disease: EEG analysis based on limited penetrable visibility graph and phase space method,” *Physica A: Statistical Mechanics and its Applications,* vol. 460, pp. 174-187, 2016.

[15]	T. Wenhart, R. A. I. Bethlehem, S. Baron-Cohen, and E. Altenmüller, “Autistic traits, resting-state connectivity, and absolute pitch in professional musicians: shared and distinct neural features,” *Mol Autism,* vol. 10, pp. 20, 2019.

[16]	Y. Dang, Y. Wang, X. Xia, Y. Yang, Y. Bai, J. Zhang, and J. He, “Deep brain stimulation improves electroencephalogram functional connectivity of patients with minimally conscious state,” *CNS Neurosci Ther,* vol. 29, no. 1, pp. 344-353, Jan, 2023.

[17]	V. D. Blondel, J.-L. Guillaume, R. Lambiotte, and E. Lefebvre, “Fast unfolding of communities in large networks,” *Journal of statistical mechanics: theory and experiment,* vol. 2008, no. 10, pp. P10008, 2008.

[18]	R. Lambiotte, J.-C. Delvenne, and M. Barahona, “Laplacian dynamics and multiscale modular structure in networks,” *arXiv preprint arXiv:0812.1770*, 2008.
