# Supplementary material for: Muscle synergies and functional network organization underlying Tai Chi movements: a pilot cross-Sectional study towards a personalized Neuromechanical Baseline
Source: Front Bioeng Biotechnol. 2026 Sep 18;14:1928241. doi: 10.3389/fbioe.2026.1928241 (PMC13631746; doi:10.3389/fbioe.2026.1928241)
Supplement: Supplementary file 2 [file Supplementaryfile2.docx]

**Supplementary Material 2.** The muscle synergy analysis results for other movements.

Supplementary Fig. 1. Muscle synergy patterns during the "Commencing Form" movement. Muscle synergy structures are presented as mean ± standard deviation. DEL: deltoid; BB: biceps brachii; TB: triceps brachii; ECRL: extensor carpi radialis longus; RF_R: rectus femoris (right); BF: biceps femoris; GC: gastrocnemius; TA: tibialis anterior. Yellow bars represent left-side muscles, and blue bars represent right-side muscles. If the muscle weightings exceed 0.1, it indicates that the muscle plays a significant role in contributing to the movement execution.

Supplementary Fig. 2. Muscle synergy patterns during the " Parting the Wild Horse’s Mane" movement. Muscle synergy structures are presented as mean ± standard deviation. DEL: deltoid; BB: biceps brachii; TB: triceps brachii; ECRL: extensor carpi radialis longus; RF_R: rectus femoris (right); BF: biceps femoris; GC: gastrocnemius; TA: tibialis anterior. Yellow bars represent left-side muscles, and blue bars represent right-side muscles. If the muscle weightings exceed 0.1, it indicates that the muscle plays a significant role in contributing to the movement execution.

Supplementary Fig. 3. Muscle synergy patterns during the " White Crane Spreads Its Wings" movement. Muscle synergy structures are presented as mean ± standard deviation. DEL: deltoid; BB: biceps brachii; TB: triceps brachii; ECRL: extensor carpi radialis longus; RF_R: rectus femoris (right); BF: biceps femoris; GC: gastrocnemius; TA: tibialis anterior. Yellow bars represent left-side muscles, and blue bars represent right-side muscles. If the muscle weightings exceed 0.1, it indicates that the muscle plays a significant role in contributing to the movement execution.

Supplementary Fig. 4. Muscle synergy patterns during the " Brush Knee and Twist Step" movement. Muscle synergy structures are presented as mean ± standard deviation. DEL: deltoid; BB: biceps brachii; TB: triceps brachii; ECRL: extensor carpi radialis longus; RF_R: rectus femoris (right); BF: biceps femoris; GC: gastrocnemius; TA: tibialis anterior. Yellow bars represent left-side muscles, and blue bars represent right-side muscles. If the muscle weightings exceed 0.1, it indicates that the muscle plays a significant role in contributing to the movement execution.

Supplementary Fig. 5. Muscle synergy patterns during the " Playing the Lute " movement. Muscle synergy structures are presented as mean ± standard deviation. DEL: deltoid; BB: biceps brachii; TB: triceps brachii; ECRL: extensor carpi radialis longus; RF_R: rectus femoris (right); BF: biceps femoris; GC: gastrocnemius; TA: tibialis anterior. Yellow bars represent left-side muscles, and blue bars represent right-side muscles. If the muscle weightings exceed 0.1, it indicates that the muscle plays a significant role in contributing to the movement execution.

Supplementary Fig. 6. Muscle synergy patterns during the "Repulse Monkey" movement. Muscle synergy structures are presented as mean ± standard deviation. DEL: deltoid; BB: biceps brachii; TB: triceps brachii; ECRL: extensor carpi radialis longus; RF_R: rectus femoris (right); BF: biceps femoris; GC: gastrocnemius; TA: tibialis anterior. Yellow bars represent left-side muscles, and blue bars represent right-side muscles. If the muscle weightings exceed 0.1, it indicates that the muscle plays a significant role in contributing to the movement execution.

Supplementary Fig. 7. Muscle synergy patterns during the " Grasp the Bird’s Tail (Left Side)" movement. Muscle synergy structures are presented as mean ± standard deviation. DEL: deltoid; BB: biceps brachii; TB: triceps brachii; ECRL: extensor carpi radialis longus; RF_R: rectus femoris (right); BF: biceps femoris; GC: gastrocnemius; TA: tibialis anterior. Yellow bars represent left-side muscles, and blue bars represent right-side muscles. If the muscle weightings exceed 0.1, it indicates that the muscle plays a significant role in contributing to the movement execution.

Supplementary Fig. 8. Muscle synergy patterns during the " Grasp the Bird’s Tail (Right Side)" movement. Muscle synergy structures are presented as mean ± standard deviation. DEL: deltoid; BB: biceps brachii; TB: triceps brachii; ECRL: extensor carpi radialis longus; RF_R: rectus femoris (right); BF: biceps femoris; GC: gastrocnemius; TA: tibialis anterior. Yellow bars represent left-side muscles, and blue bars represent right-side muscles. If the muscle weightings exceed 0.1, it indicates that the muscle plays a significant role in contributing to the movement execution.

Supplementary Fig. 9. Muscle synergy patterns during the " Single Whip 1" movement. Muscle synergy structures are presented as mean ± standard deviation. DEL: deltoid; BB: biceps brachii; TB: triceps brachii; ECRL: extensor carpi radialis longus; RF_R: rectus femoris (right); BF: biceps femoris; GC: gastrocnemius; TA: tibialis anterior. Yellow bars represent left-side muscles, and blue bars represent right-side muscles. If the muscle weightings exceed 0.1, it indicates that the muscle plays a significant role in contributing to the movement execution.

Supplementary Fig. 10. Muscle synergy patterns during the " Wave Hands Like Clouds" movement. Muscle synergy structures are presented as mean ± standard deviation. DEL: deltoid; BB: biceps brachii; TB: triceps brachii; ECRL: extensor carpi radialis longus; RF_R: rectus femoris (right); BF: biceps femoris; GC: gastrocnemius; TA: tibialis anterior. Yellow bars represent left-side muscles, and blue bars represent right-side muscles. If the muscle weightings exceed 0.1, it indicates that the muscle plays a significant role in contributing to the movement execution.

Supplementary Fig. 11. Muscle synergy patterns during the " Single Whip 2" movement. Muscle synergy structures are presented as mean ± standard deviation. DEL: deltoid; BB: biceps brachii; TB: triceps brachii; ECRL: extensor carpi radialis longus; RF_R: rectus femoris (right); BF: biceps femoris; GC: gastrocnemius; TA: tibialis anterior. Yellow bars represent left-side muscles, and blue bars represent right-side muscles. If the muscle weightings exceed 0.1, it indicates that the muscle plays a significant role in contributing to the movement execution.

Supplementary Fig. 12. Muscle synergy patterns during the "High Pat on Horse" movement. Muscle synergy structures are presented as mean ± standard deviation. DEL: deltoid; BB: biceps brachii; TB: triceps brachii; ECRL: extensor carpi radialis longus; RF_R: rectus femoris (right); BF: biceps femoris; GC: gastrocnemius; TA: tibialis anterior. Yellow bars represent left-side muscles, and blue bars represent right-side muscles. If the muscle weightings exceed 0.1, it indicates that the muscle plays a significant role in contributing to the movement execution.

Supplementary Fig. 13. Muscle synergy patterns during the "Right Heel Kick" movement. Muscle synergy structures are presented as mean ± standard deviation. DEL: deltoid; BB: biceps brachii; TB: triceps brachii; ECRL: extensor carpi radialis longus; RF_R: rectus femoris (right); BF: biceps femoris; GC: gastrocnemius; TA: tibialis anterior. Yellow bars represent left-side muscles, and blue bars represent right-side muscles. If the muscle weightings exceed 0.1, it indicates that the muscle plays a significant role in contributing to the movement execution.

Supplementary Fig. 14. Muscle synergy patterns during the "Strike to Ears with Both Fists" movement. Muscle synergy structures are presented as mean ± standard deviation. DEL: deltoid; BB: biceps brachii; TB: triceps brachii; ECRL: extensor carpi radialis longus; RF_R: rectus femoris (right); BF: biceps femoris; GC: gastrocnemius; TA: tibialis anterior. Yellow bars represent left-side muscles, and blue bars represent right-side muscles. If the muscle weightings exceed 0.1, it indicates that the muscle plays a significant role in contributing to the movement execution.

Supplementary Fig. 15. Muscle synergy patterns during the "Turn and Left Heel Kick" movement. Muscle synergy structures are presented as mean ± standard deviation. DEL: deltoid; BB: biceps brachii; TB: triceps brachii; ECRL: extensor carpi radialis longus; RF_R: rectus femoris (right); BF: biceps femoris; GC: gastrocnemius; TA: tibialis anterior. Yellow bars represent left-side muscles, and blue bars represent right-side muscles. If the muscle weightings exceed 0.1, it indicates that the muscle plays a significant role in contributing to the movement execution.

Supplementary Fig. 16. Muscle synergy patterns during the "Left Lower Body and Stand on One Leg" movement. Muscle synergy structures are presented as mean ± standard deviation. DEL: deltoid; BB: biceps brachii; TB: triceps brachii; ECRL: extensor carpi radialis longus; RF_R: rectus femoris (right); BF: biceps femoris; GC: gastrocnemius; TA: tibialis anterior. Yellow bars represent left-side muscles, and blue bars represent right-side muscles. If the muscle weightings exceed 0.1, it indicates that the muscle plays a significant role in contributing to the movement execution.

Supplementary Fig. 17. Muscle synergy patterns during the "Right Lower Body and Stand on One Leg" movement. Muscle synergy structures are presented as mean ± standard deviation. DEL: deltoid; BB: biceps brachii; TB: triceps brachii; ECRL: extensor carpi radialis longus; RF_R: rectus femoris (right); BF: biceps femoris; GC: gastrocnemius; TA: tibialis anterior. Yellow bars represent left-side muscles, and blue bars represent right-side muscles. If the muscle weightings exceed 0.1, it indicates that the muscle plays a significant role in contributing to the movement execution.

Supplementary Fig. 18. Muscle synergy patterns during the "Shuttle Back and Forth" movement. Muscle synergy structures are presented as mean ± standard deviation. DEL: deltoid; BB: biceps brachii; TB: triceps brachii; ECRL: extensor carpi radialis longus; RF_R: rectus femoris (right); BF: biceps femoris; GC: gastrocnemius; TA: tibialis anterior. Yellow bars represent left-side muscles, and blue bars represent right-side muscles. If the muscle weightings exceed 0.1, it indicates that the muscle plays a significant role in contributing to the movement execution.

Supplementary Fig. 19. Muscle synergy patterns during the "Needle at Sea Bottom" movement. Muscle synergy structures are presented as mean ± standard deviation. DEL: deltoid; BB: biceps brachii; TB: triceps brachii; ECRL: extensor carpi radialis longus; RF_R: rectus femoris (right); BF: biceps femoris; GC: gastrocnemius; TA: tibialis anterior. Yellow bars represent left-side muscles, and blue bars represent right-side muscles. If the muscle weightings exceed 0.1, it indicates that the muscle plays a significant role in contributing to the movement execution.

Supplementary Fig. 20. Muscle synergy patterns during the "Fan Through the Back" movement. Muscle synergy structures are presented as mean ± standard deviation. DEL: deltoid; BB: biceps brachii; TB: triceps brachii; ECRL: extensor carpi radialis longus; RF_R: rectus femoris (right); BF: biceps femoris; GC: gastrocnemius; TA: tibialis anterior. Yellow bars represent left-side muscles, and blue bars represent right-side muscles. If the muscle weightings exceed 0.1, it indicates that the muscle plays a significant role in contributing to the movement execution.

Supplementary Fig. 21. Muscle synergy patterns during the "Turn, Deflect, Parry, and Punch" movement. Muscle synergy structures are presented as mean ± standard deviation. DEL: deltoid; BB: biceps brachii; TB: triceps brachii; ECRL: extensor carpi radialis longus; RF_R: rectus femoris (right); BF: biceps femoris; GC: gastrocnemius; TA: tibialis anterior. Yellow bars represent left-side muscles, and blue bars represent right-side muscles. If the muscle weightings exceed 0.1, it indicates that the muscle plays a significant role in contributing to the movement execution.

Supplementary Fig. 22. Muscle synergy patterns during the "Apparent Closing Up" movement. Muscle synergy structures are presented as mean ± standard deviation. DEL: deltoid; BB: biceps brachii; TB: triceps brachii; ECRL: extensor carpi radialis longus; RF_R: rectus femoris (right); BF: biceps femoris; GC: gastrocnemius; TA: tibialis anterior. Yellow bars represent left-side muscles, and blue bars represent right-side muscles. If the muscle weightings exceed 0.1, it indicates that the muscle plays a significant role in contributing to the movement execution.

Supplementary Fig. 23. Muscle synergy patterns during the "Cross Hands" movement. Muscle synergy structures are presented as mean ± standard deviation. DEL: deltoid; BB: biceps brachii; TB: triceps brachii; ECRL: extensor carpi radialis longus; RF_R: rectus femoris (right); BF: biceps femoris; GC: gastrocnemius; TA: tibialis anterior. Yellow bars represent left-side muscles, and blue bars represent right-side muscles. If the muscle weightings exceed 0.1, it indicates that the muscle plays a significant role in contributing to the movement execution.

Supplementary Fig. 24. Muscle synergy patterns during the "Closing Form" movement. Muscle synergy structures are presented as mean ± standard deviation. DEL: deltoid; BB: biceps brachii; TB: triceps brachii; ECRL: extensor carpi radialis longus; RF_R: rectus femoris (right); BF: biceps femoris; GC: gastrocnemius; TA: tibialis anterior. Yellow bars represent left-side muscles, and blue bars represent right-side muscles. If the muscle weightings exceed 0.1, it indicates that the muscle plays a significant role in contributing to the movement execution.
