# Supplementary material for: Muscle synergies and functional network organization underlying Tai Chi movements: a pilot cross-Sectional study towards a personalized Neuromechanical Baseline
Source: Front Bioeng Biotechnol. 2026 Sep 18;14:1928241. doi: 10.3389/fbioe.2026.1928241 (PMC13631746; doi:10.3389/fbioe.2026.1928241)
Supplement: Supplementary file 3 [file Supplementaryfile3.docx]

**Supplementary Material 3.** Table 1. Muscle pairs with high normalized mutual information (NMI) in 24 Tai Chi movements.

| Movement | NMI Value | Muscle Pair |
| --- | --- | --- |
| Commencing Form | 0.55 | TA_L - GC_R |
|  | 0.49 | BB_R - BB_L |
|  | 0.46 | BB_L - DEL_R |
|  | 0.45 | ECRL_L - TB_R |
|  | 0.44 | TB_L - BB_R |
| Parting the Wild Horse’s Mane | 0.49 | GC_L - BF_R |
|  | 0.44 | TA_L - GC_R |
|  | 0.42 | GC_R - GC_L |
|  | 0.41 | BF_R - BF_L |
|  | 0.39 | BB_R - BB_L |
| White Crane Spreads Its Wings | 0.51 | BB_L - DEL_R |
|  | 0.43 | BB_R - BB_L |
|  | 0.39 | BB_R - DEL_R |
|  | 0.38 | TA_L - GC_R |
|  | 0.37 | TB_L - BB_R |
| Brush Knee and Twist Step | 0.39 | BB_R - BB_L |
|  | 0.38 | GC_L - BF_R |
|  | 0.38 | TA_L - GC_R |
|  | 0.37 | BB_L - DEL_R |
|  | 0.31 | GC_R - GC_L |
| Playing the Lute | 0.50 | BB_L - DEL_R |
|  | 0.47 | BB_R - BB_L |
|  | 0.43 | TA_L - GC_R |
|  | 0.41 | BB_R - DEL_R |
|  | 0.41 | GC_L - BF_R |
| Repulse Monkey | 0.42 | BB_L - DEL_R |
|  | 0.36 | BB_R - BB_L |
|  | 0.34 | TA_L - GC_R |
|  | 0.31 | GC_L - BF_R |
|  | 0.31 | BB_R - DEL_R |
| Grasp the Bird’s Tail (Left Side) | 0.47 | BB_R - BB_L |
|  | 0.47 | BB_L - DEL_R |
|  | 0.38 | BB_R - DEL_R |
|  | 0.37 | TA_L - GC_R |
|  | 0.36 | TB_L - BB_R |
| Grasp the Bird’s Tail (Right Side) | 0.40 | TA_L - GC_R |
|  | 0.37 | BB_L - DEL_R |
|  | 0.36 | GC_L - BF_R |

Continued Table 1. Muscle pairs with high normalized mutual information (NMI) in 24 Tai Chi movements.

| Movement | NMI Value | Muscle Pair |
| --- | --- | --- |
|  | 0.35 | BB_R - BB_L |
|  | 0.32 | GC_R - GC_L |
| Single Whip | 0.47 | BB_L - DEL_R |
|  | 0.46 | BB_R - BB_L |
|  | 0.39 | BB_R - DEL_R |
|  | 0.38 | TB_L - BB_R |
|  | 0.36 | TA_L - GC_R |
| Wave Hands Like Clouds | 0.40 | BB_L - DEL_R |
|  | 0.39 | BB_R - BB_L |
|  | 0.37 | GC_L - BF_R |
|  | 0.36 | TA_L - GC_R |
|  | 0.35 | GC_R - GC_L |
| Single Whip | 0.50 | BB_R - BB_L |
|  | 0.49 | BB_L - DEL_R |
|  | 0.43 | BB_R - DEL_R |
|  | 0.43 | TA_L - GC_R |
|  | 0.41 | GC_R - GC_L |
| High Pat on Horse | 0.49 | BB_L - DEL_R |
|  | 0.43 | BB_R - BB_L |
|  | 0.41 | TA_L - GC_R |
|  | 0.39 | GC_R - GC_L |
|  | 0.39 | BB_R - DEL_R |
| Right Heel Kick | 0.51 | BB_R - BB_L |
|  | 0.48 | BB_L - DEL_R |
|  | 0.43 | BB_R - DEL_R |
|  | 0.41 | TA_L - GC_R |
|  | 0.37 | GC_L - BF_R |
| Strike to Ears with Both Fists | 0.46 | BB_R - BB_L |
|  | 0.46 | RF_L - ECRL_R |
|  | 0.43 | BB_L - DEL_R |
|  | 0.41 | TA_L - GC_R |
|  | 0.38 | BB_R - DEL_R |
| Turn and Left Heel Kick | 0.55 | TA_L - GC_R |
|  | 0.49 | GC_L - BF_R |
|  | 0.49 | GC_R - GC_L |
|  | 0.43 | BF_R - BF_L |
|  | 0.41 | GC_R - BF_R |
| Left Lower Body and Stand on One Leg | 0.43 | BB_L - DEL_R |
|  | 0.41 | BB_R - BB_L |

Continued Table 1. Muscle pairs with high normalized mutual information (NMI) in 24 Tai Chi movements.

| Movement | NMI Value | Muscle Pair |
| --- | --- | --- |
|  | 0.39 | TA_L - GC_R |
|  | 0.36 | BF_R - BF_L |
|  | 0.35 | RF_R - RF_L |
| Right Lower Body and Stand on One Leg | 0.40 | GC_L - BF_R |
|  | 0.38 | GC_R - GC_L |
|  | 0.37 | BB_R - BB_L |
|  | 0.37 | BB_L - DEL_R |
|  | 0.36 | TA_L - GC_R |
| Shuttle Back and Forth | 0.40 | BB_R - BB_L |
|  | 0.38 | BB_L - DEL_R |
|  | 0.37 | TA_L - GC_R |
|  | 0.33 | GC_L - BF_R |
|  | 0.31 | BB_R - DEL_R |
| Needle at Sea Bottom | 0.47 | BB_L - DEL_R |
|  | 0.43 | TA_L - GC_R |
|  | 0.43 | BB_R - BB_L |
|  | 0.40 | BB_R - DEL_R |
|  | 0.39 | GC_R - GC_L |
| Fan Through the Back | 0.46 | BB_R - BB_L |
|  | 0.45 | BB_L - DEL_R |
|  | 0.44 | TA_L - GC_R |
|  | 0.40 | RF_R - RF_L |
|  | 0.38 | BB_R - DEL_R |
| Turn, Deflect, Parry, and Punch | 0.40 | TA_L - GC_R |
|  | 0.39 | BB_L - DEL_R |
|  | 0.39 | BB_R - BB_L |
|  | 0.34 | BF_R - BF_L |
|  | 0.34 | GC_L - BF_R |
| Apparent Closing Up | 0.49 | BB_L - DEL_R |
|  | 0.46 | BB_R - BB_L |
|  | 0.45 | TA_L - GC_R |
|  | 0.40 | BB_R - DEL_R |
|  | 0.40 | TB_L - BB_R |
| Cross Hands | 0.45 | TA_L - GC_R |
|  | 0.45 | BB_L - DEL_R |
|  | 0.45 | BB_R - BB_L |
|  | 0.44 | GC_L - BF_R |
|  | 0.44 | GC_R - GC_L |
| Closing Form | 0.52 | TA_L - GC_R |

Continued Table 1. Muscle pairs with high normalized mutual information (NMI) in 24 Tai Chi movements.

| Movement | NMI Value | Muscle Pair |
| --- | --- | --- |
|  | 0.46 | BB_L - DEL_R |
|  | 0.45 | BF_L - RF_R |
|  | 0.43 | BB_R - BB_L |
|  | 0.43 | BF_R - BF_L |

DEL_L, Left Deltoid; DEL_R, Right Deltoid; BB, Biceps Brachii; TB, Triceps Brachii; ECRL, Extensor Carpi Radialis Longus; RF_R, Right Rectus Femoris; BF, Biceps Femoris; GC, Gastrocnemius; TA, Tibialis Anterior.
