# Supplementary material for: Muscle synergies and functional network organization underlying Tai Chi movements: a pilot cross-Sectional study towards a personalized Neuromechanical Baseline
Source: Front Bioeng Biotechnol. 2026 Sep 18;14:1928241. doi: 10.3389/fbioe.2026.1928241 (PMC13631746; doi:10.3389/fbioe.2026.1928241)
Supplement: Supplementary file 4 [file Supplementaryfile4.docx]

**Supplementary Material 4.** NMI visualizations for the other movements.

Supplementary Fig. 1. Muscle coupling intensity distribution of Commencing Form. Higher NMI values indicate stronger correlation, shown by colors closer to deep yellow, while colors near deep blue represent weaker correlations. C1: left deltoid, C2: right deltoid, C3: left biceps brachii, C4: right biceps brachii, C5: left triceps brachii, C6: right triceps brachii, C7: left extensor carpi radialis longus, C8: right extensor carpi radialis longus, C9: left rectus femoris, C10: right rectus femoris, C11: left biceps femoris, C12: right biceps femoris, C13: left gastrocnemius, C14: right gastrocnemius, C15: left tibialis anterior, C16: right tibialis anterior.

Supplementary Fig. 2. Muscle coupling intensity distribution of Parting the Wild Horse’s Mane. Higher NMI values indicate stronger correlation, shown by colors closer to deep yellow, while colors near deep blue represent weaker correlations. C1: left deltoid, C2: right deltoid, C3: left biceps brachii, C4: right biceps brachii, C5: left triceps brachii, C6: right triceps brachii, C7: left extensor carpi radialis longus, C8: right extensor carpi radialis longus, C9: left rectus femoris, C10: right rectus femoris, C11: left biceps femoris, C12: right biceps femoris, C13: left gastrocnemius, C14: right gastrocnemius, C15: left tibialis anterior, C16: right tibialis anterior.

Supplementary Fig. 3. Muscle coupling intensity distribution of White Crane Spreads Its Wings. Higher NMI values indicate stronger correlation, shown by colors closer to deep yellow, while colors near deep blue represent weaker correlations. C1: left deltoid, C2: right deltoid, C3: left biceps brachii, C4: right biceps brachii, C5: left triceps brachii, C6: right triceps brachii, C7: left extensor carpi radialis longus, C8: right extensor carpi radialis longus, C9: left rectus femoris, C10: right rectus femoris, C11: left biceps femoris, C12: right biceps femoris, C13: left gastrocnemius, C14: right gastrocnemius, C15: left tibialis anterior, C16: right tibialis anterior.

Supplementary Fig. 4. Muscle coupling intensity distribution of Brush Knee and Twist Step. Higher NMI values indicate stronger correlation, shown by colors closer to deep yellow, while colors near deep blue represent weaker correlations. C1: left deltoid, C2: right deltoid, C3: left biceps brachii, C4: right biceps brachii, C5: left triceps brachii, C6: right triceps brachii, C7: left extensor carpi radialis longus, C8: right extensor carpi radialis longus, C9: left rectus femoris, C10: right rectus femoris, C11: left biceps femoris, C12: right biceps femoris, C13: left gastrocnemius, C14: right gastrocnemius, C15: left tibialis anterior, C16: right tibialis anterior.

Supplementary Fig. 5. Muscle coupling intensity distribution of Playing the Lute. Higher NMI values indicate stronger correlation, shown by colors closer to deep yellow, while colors near deep blue represent weaker correlations. C1: left deltoid, C2: right deltoid, C3: left biceps brachii, C4: right biceps brachii, C5: left triceps brachii, C6: right triceps brachii, C7: left extensor carpi radialis longus, C8: right extensor carpi radialis longus, C9: left rectus femoris, C10: right rectus femoris, C11: left biceps femoris, C12: right biceps femoris, C13: left gastrocnemius, C14: right gastrocnemius, C15: left tibialis anterior, C16: right tibialis anterior.

Supplementary Fig. 6. Muscle coupling intensity distribution of Repulse Monkey. Higher NMI values indicate stronger correlation, shown by colors closer to deep yellow, while colors near deep blue represent weaker correlations. C1: left deltoid, C2: right deltoid, C3: left biceps brachii, C4: right biceps brachii, C5: left triceps brachii, C6: right triceps brachii, C7: left extensor carpi radialis longus, C8: right extensor carpi radialis longus, C9: left rectus femoris, C10: right rectus femoris, C11: left biceps femoris, C12: right biceps femoris, C13: left gastrocnemius, C14: right gastrocnemius, C15: left tibialis anterior, C16: right tibialis anterior.

Supplementary Fig. 7. Muscle coupling intensity distribution of Grasp the Bird’s Tail (Left Side). Higher NMI values indicate stronger correlation, shown by colors closer to deep yellow, while colors near deep blue represent weaker correlations. C1: left deltoid, C2: right deltoid, C3: left biceps brachii, C4: right biceps brachii, C5: left triceps brachii, C6: right triceps brachii, C7: left extensor carpi radialis longus, C8: right extensor carpi radialis longus, C9: left rectus femoris, C10: right rectus femoris, C11: left biceps femoris, C12: right biceps femoris, C13: left gastrocnemius, C14: right gastrocnemius, C15: left tibialis anterior, C16: right tibialis anterior.

Supplementary Fig. 8. Muscle coupling intensity distribution of Grasp the Bird’s Tail (Right Side). Higher NMI values indicate stronger correlation, shown by colors closer to deep yellow, while colors near deep blue represent weaker correlations. C1: left deltoid, C2: right deltoid, C3: left biceps brachii, C4: right biceps brachii, C5: left triceps brachii, C6: right triceps brachii, C7: left extensor carpi radialis longus, C8: right extensor carpi radialis longus, C9: left rectus femoris, C10: right rectus femoris, C11: left biceps femoris, C12: right biceps femoris, C13: left gastrocnemius, C14: right gastrocnemius, C15: left tibialis anterior, C16: right tibialis anterior.

Supplementary Fig. 9. Muscle coupling intensity distribution of Single Whip 1. Higher NMI values indicate stronger correlation, shown by colors closer to deep yellow, while colors near deep blue represent weaker correlations. C1: left deltoid, C2: right deltoid, C3: left biceps brachii, C4: right biceps brachii, C5: left triceps brachii, C6: right triceps brachii, C7: left extensor carpi radialis longus, C8: right extensor carpi radialis longus, C9: left rectus femoris, C10: right rectus femoris, C11: left biceps femoris, C12: right biceps femoris, C13: left gastrocnemius, C14: right gastrocnemius, C15: left tibialis anterior, C16: right tibialis anterior.

Supplementary Fig. 10. Muscle coupling intensity distribution of Wave Hands Like Clouds. Higher NMI values indicate stronger correlation, shown by colors closer to deep yellow, while colors near deep blue represent weaker correlations. C1: left deltoid, C2: right deltoid, C3: left biceps brachii, C4: right biceps brachii, C5: left triceps brachii, C6: right triceps brachii, C7: left extensor carpi radialis longus, C8: right extensor carpi radialis longus, C9: left rectus femoris, C10: right rectus femoris, C11: left biceps femoris, C12: right biceps femoris, C13: left gastrocnemius, C14: right gastrocnemius, C15: left tibialis anterior, C16: right tibialis anterior.

Supplementary Fig. 11. Muscle coupling intensity distribution of Single Whip 2. Higher NMI values indicate stronger correlation, shown by colors closer to deep yellow, while colors near deep blue represent weaker correlations. C1: left deltoid, C2: right deltoid, C3: left biceps brachii, C4: right biceps brachii, C5: left triceps brachii, C6: right triceps brachii, C7: left extensor carpi radialis longus, C8: right extensor carpi radialis longus, C9: left rectus femoris, C10: right rectus femoris, C11: left biceps femoris, C12: right biceps femoris, C13: left gastrocnemius, C14: right gastrocnemius, C15: left tibialis anterior, C16: right tibialis anterior.

Supplementary Fig. 12. Muscle coupling intensity distribution of High Pat on Horse. Higher NMI values indicate stronger correlation, shown by colors closer to deep yellow, while colors near deep blue represent weaker correlations. C1: left deltoid, C2: right deltoid, C3: left biceps brachii, C4: right biceps brachii, C5: left triceps brachii, C6: right triceps brachii, C7: left extensor carpi radialis longus, C8: right extensor carpi radialis longus, C9: left rectus femoris, C10: right rectus femoris, C11: left biceps femoris, C12: right biceps femoris, C13: left gastrocnemius, C14: right gastrocnemius, C15: left tibialis anterior, C16: right tibialis anterior.

Supplementary Fig. 13. Muscle coupling intensity distribution of Right Heel Kick. Higher NMI values indicate stronger correlation, shown by colors closer to deep yellow, while colors near deep blue represent weaker correlations. C1: left deltoid, C2: right deltoid, C3: left biceps brachii, C4: right biceps brachii, C5: left triceps brachii, C6: right triceps brachii, C7: left extensor carpi radialis longus, C8: right extensor carpi radialis longus, C9: left rectus femoris, C10: right rectus femoris, C11: left biceps femoris, C12: right biceps femoris, C13: left gastrocnemius, C14: right gastrocnemius, C15: left tibialis anterior, C16: right tibialis anterior.

Supplementary Fig. 14. Muscle coupling intensity distribution of Strike to Ears with Both Fists. Higher NMI values indicate stronger correlation, shown by colors closer to deep yellow, while colors near deep blue represent weaker correlations. C1: left deltoid, C2: right deltoid, C3: left biceps brachii, C4: right biceps brachii, C5: left triceps brachii, C6: right triceps brachii, C7: left extensor carpi radialis longus, C8: right extensor carpi radialis longus, C9: left rectus femoris, C10: right rectus femoris, C11: left biceps femoris, C12: right biceps femoris, C13: left gastrocnemius, C14: right gastrocnemius, C15: left tibialis anterior, C16: right tibialis anterior.

Supplementary Fig. 15. Muscle coupling intensity distribution of Turn and Left Heel Kick. Higher NMI values indicate stronger correlation, shown by colors closer to deep yellow, while colors near deep blue represent weaker correlations. C1: left deltoid, C2: right deltoid, C3: left biceps brachii, C4: right biceps brachii, C5: left triceps brachii, C6: right triceps brachii, C7: left extensor carpi radialis longus, C8: right extensor carpi radialis longus, C9: left rectus femoris, C10: right rectus femoris, C11: left biceps femoris, C12: right biceps femoris, C13: left gastrocnemius, C14: right gastrocnemius, C15: left tibialis anterior, C16: right tibialis anterior.

Supplementary Fig. 16. Muscle coupling intensity distribution of Left Lower Body and Stand on One Leg. Higher NMI values indicate stronger correlation, shown by colors closer to deep yellow, while colors near deep blue represent weaker correlations. C1: left deltoid, C2: right deltoid, C3: left biceps brachii, C4: right biceps brachii, C5: left triceps brachii, C6: right triceps brachii, C7: left extensor carpi radialis longus, C8: right extensor carpi radialis longus, C9: left rectus femoris, C10: right rectus femoris, C11: left biceps femoris, C12: right biceps femoris, C13: left gastrocnemius, C14: right gastrocnemius, C15: left tibialis anterior, C16: right tibialis anterior.

Supplementary Fig. 17. Muscle coupling intensity distribution of Right Lower Body and Stand on One Leg. Higher NMI values indicate stronger correlation, shown by colors closer to deep yellow, while colors near deep blue represent weaker correlations. C1: left deltoid, C2: right deltoid, C3: left biceps brachii, C4: right biceps brachii, C5: left triceps brachii, C6: right triceps brachii, C7: left extensor carpi radialis longus, C8: right extensor carpi radialis longus, C9: left rectus femoris, C10: right rectus femoris, C11: left biceps femoris, C12: right biceps femoris, C13: left gastrocnemius, C14: right gastrocnemius, C15: left tibialis anterior, C16: right tibialis anterior.

Supplementary Fig. 18. Muscle coupling intensity distribution of Shuttle Back and Forth. Higher NMI values indicate stronger correlation, shown by colors closer to deep yellow, while colors near deep blue represent weaker correlations. C1: left deltoid, C2: right deltoid, C3: left biceps brachii, C4: right biceps brachii, C5: left triceps brachii, C6: right triceps brachii, C7: left extensor carpi radialis longus, C8: right extensor carpi radialis longus, C9: left rectus femoris, C10: right rectus femoris, C11: left biceps femoris, C12: right biceps femoris, C13: left gastrocnemius, C14: right gastrocnemius, C15: left tibialis anterior, C16: right tibialis anterior.

Supplementary Fig. 19. Muscle coupling intensity distribution of Needle at Sea Bottom. Higher NMI values indicate stronger correlation, shown by colors closer to deep yellow, while colors near deep blue represent weaker correlations. C1: left deltoid, C2: right deltoid, C3: left biceps brachii, C4: right biceps brachii, C5: left triceps brachii, C6: right triceps brachii, C7: left extensor carpi radialis longus, C8: right extensor carpi radialis longus, C9: left rectus femoris, C10: right rectus femoris, C11: left biceps femoris, C12: right biceps femoris, C13: left gastrocnemius, C14: right gastrocnemius, C15: left tibialis anterior, C16: right tibialis anterior.

Supplementary Fig. 20. Muscle coupling intensity distribution of Fan Through the Back. Higher NMI values indicate stronger correlation, shown by colors closer to deep yellow, while colors near deep blue represent weaker correlations. C1: left deltoid, C2: right deltoid, C3: left biceps brachii, C4: right biceps brachii, C5: left triceps brachii, C6: right triceps brachii, C7: left extensor carpi radialis longus, C8: right extensor carpi radialis longus, C9: left rectus femoris, C10: right rectus femoris, C11: left biceps femoris, C12: right biceps femoris, C13: left gastrocnemius, C14: right gastrocnemius, C15: left tibialis anterior, C16: right tibialis anterior.

Supplementary Fig. 21. Muscle coupling intensity distribution of Turn, Deflect, Parry, and Punch. Higher NMI values indicate stronger correlation, shown by colors closer to deep yellow, while colors near deep blue represent weaker correlations. C1: left deltoid, C2: right deltoid, C3: left biceps brachii, C4: right biceps brachii, C5: left triceps brachii, C6: right triceps brachii, C7: left extensor carpi radialis longus, C8: right extensor carpi radialis longus, C9: left rectus femoris, C10: right rectus femoris, C11: left biceps femoris, C12: right biceps femoris, C13: left gastrocnemius, C14: right gastrocnemius, C15: left tibialis anterior, C16: right tibialis anterior.

Supplementary Fig. 22. Muscle coupling intensity distribution of Apparent Closing Up. Higher NMI values indicate stronger correlation, shown by colors closer to deep yellow, while colors near deep blue represent weaker correlations. C1: left deltoid, C2: right deltoid, C3: left biceps brachii, C4: right biceps brachii, C5: left triceps brachii, C6: right triceps brachii, C7: left extensor carpi radialis longus, C8: right extensor carpi radialis longus, C9: left rectus femoris, C10: right rectus femoris, C11: left biceps femoris, C12: right biceps femoris, C13: left gastrocnemius, C14: right gastrocnemius, C15: left tibialis anterior, C16: right tibialis anterior.

Supplementary Fig. 23. Muscle coupling intensity distribution of Cross Hands. Higher NMI values indicate stronger correlation, shown by colors closer to deep yellow, while colors near deep blue represent weaker correlations. C1: left deltoid, C2: right deltoid, C3: left biceps brachii, C4: right biceps brachii, C5: left triceps brachii, C6: right triceps brachii, C7: left extensor carpi radialis longus, C8: right extensor carpi radialis longus, C9: left rectus femoris, C10: right rectus femoris, C11: left biceps femoris, C12: right biceps femoris, C13: left gastrocnemius, C14: right gastrocnemius, C15: left tibialis anterior, C16: right tibialis anterior.

Supplementary Fig. 24. Muscle coupling intensity distribution of Closing Form. Higher NMI values indicate stronger correlation, shown by colors closer to deep yellow, while colors near deep blue represent weaker correlations. C1: left deltoid, C2: right deltoid, C3: left biceps brachii, C4: right biceps brachii, C5: left triceps brachii, C6: right triceps brachii, C7: left extensor carpi radialis longus, C8: right extensor carpi radialis longus, C9: left rectus femoris, C10: right rectus femoris, C11: left biceps femoris, C12: right biceps femoris, C13: left gastrocnemius, C14: right gastrocnemius, C15: left tibialis anterior, C16: right tibialis anterior.
