# Supplementary material for: Muscle synergies and functional network organization underlying Tai Chi movements: a pilot cross-Sectional study towards a personalized Neuromechanical Baseline
Source: Front Bioeng Biotechnol. 2026 Sep 18;14:1928241. doi: 10.3389/fbioe.2026.1928241 (PMC13631746; doi:10.3389/fbioe.2026.1928241)
Supplement: Supplementary file 5 [file Supplementaryfile5.docx]

**Supplementary Material 5.** The detailed results of the threshold calculations for each movement.

Supplementary Fig. 1. The detailed results of the threshold calculations for Commencing Form.

Supplementary Fig. 2. The detailed results of the threshold calculations for Parting the Wild Horse’s Mane.

Supplementary Fig. 3. The detailed results of the threshold calculations for White Crane Spreads Its Wings.

Supplementary Fig. 4. The detailed results of the threshold calculations for Brush Knee and Twist Step.

Supplementary Fig. 5. The detailed results of the threshold calculations for Playing the Lute.

Supplementary Fig. 6. The detailed results of the threshold calculations for Repulse Monkey.

Supplementary Fig. 7. The detailed results of the threshold calculations for Grasp the Bird’s Tail (Left Side).

Supplementary Fig. 8. The detailed results of the threshold calculations for Grasp the Bird’s Tail (Right Side).

Supplementary Fig. 9. The detailed results of the threshold calculations for Single Whip 1.

Supplementary Fig. 10. The detailed results of the threshold calculations for Wave Hands Like Clouds.

Supplementary Fig. 11. The detailed results of the threshold calculations for Single Whip 2.

Supplementary Fig. 12. The detailed results of the threshold calculations for High Pat on Horse.

Supplementary Fig. 13. The detailed results of the threshold calculations for Right Heel Kick.

Supplementary Fig. 14. The detailed results of the threshold calculations for Strike to Ears with Both Fists.

Supplementary Fig. 15. The detailed results of the threshold calculations for Turn and Left Heel Kick.

Supplementary Fig. 16. The detailed results of the threshold calculations for Left Lower Body and Stand on One Leg.

Supplementary Fig. 17. The detailed results of the threshold calculations for Right Lower Body and Stand on One Leg.

Supplementary Fig. 18. The detailed results of the threshold calculations for Shuttle Back and Forth.

Supplementary Fig. 19. The detailed results of the threshold calculations for Needle at Sea Bottom.

Supplementary Fig. 20. The detailed results of the threshold calculations for Fan Through the Back.

Supplementary Fig. 21. The detailed results of the threshold calculations for Turn, Deflect, Parry, and Punch.

Supplementary Fig. 22. The detailed results of the threshold calculations for Apparent Closing Up.

Supplementary Fig. 23. The detailed results of the threshold calculations for Cross Hands.

Supplementary Fig. 24. The detailed results of the threshold calculations for Closing Form.
