# Supplementary material for: Muscle synergies and functional network organization underlying Tai Chi movements: a pilot cross-Sectional study towards a personalized Neuromechanical Baseline
Source: Front Bioeng Biotechnol. 2026 Sep 18;14:1928241. doi: 10.3389/fbioe.2026.1928241 (PMC13631746; doi:10.3389/fbioe.2026.1928241)
Supplement: Supplementary file 6 [file Supplementaryfile6.docx]

**Supplementary Material 5.** Schematic diagrams of the muscle functional connectivity networks for the other movements.

Supplementary Fig. 1. Functional muscle network structure for the movement " Commencing Form ". Larger nodes indicate higher degree centrality, reflecting greater importance within the network, while smaller nodes indicate lower centrality. Thicker edges between nodes represent stronger coherence (higher mutual information), whereas thinner edges indicate weaker connections. "Community" refer to clusters of muscles with strong intragroup functional coupling; within-community connections are significantly stronger than those between communities, typically representing functionally coordinated muscle units specific to the movement pattern. DEL_L: left deltoid, DEL_R: right deltoid, BB_L: left biceps brachii, BB_R: right biceps brachii, TB_L: left triceps brachii, TB_R: right triceps brachii, ECRL_L: left extensor carpi radialis longus, ECRL_R: right extensor carpi radialis longus, RF_L: left rectus femoris, RF_R: right rectus femoris, BF_L: left biceps femoris, BF_R: right biceps femoris, GC_L: left gastrocnemius, GC_R: right gastrocnemius, TA_L: left tibialis anterior, TA_R: right tibialis anterior.

Supplementary Fig. 2. Functional muscle network structure for the movement " Parting the Wild Horse’s Mane ". Larger nodes indicate higher degree centrality, reflecting greater importance within the network, while smaller nodes indicate lower centrality. Thicker edges between nodes represent stronger coherence (higher mutual information), whereas thinner edges indicate weaker connections. "Community" refer to clusters of muscles with strong intragroup functional coupling; within-community connections are significantly stronger than those between communities, typically representing functionally coordinated muscle units specific to the movement pattern. DEL_L: left deltoid, DEL_R: right deltoid, BB_L: left biceps brachii, BB_R: right biceps brachii, TB_L: left triceps brachii, TB_R: right triceps brachii, ECRL_L: left extensor carpi radialis longus, ECRL_R: right extensor carpi radialis longus, RF_L: left rectus femoris, RF_R: right rectus femoris, BF_L: left biceps femoris, BF_R: right biceps femoris, GC_L: left gastrocnemius, GC_R: right gastrocnemius, TA_L: left tibialis anterior, TA_R: right tibialis anterior.

Supplementary Fig. 3. Functional muscle network structure for the movement " White Crane Spreads Its Wings ". Larger nodes indicate higher degree centrality, reflecting greater importance within the network, while smaller nodes indicate lower centrality. Thicker edges between nodes represent stronger coherence (higher mutual information), whereas thinner edges indicate weaker connections. "Community" refer to clusters of muscles with strong intragroup functional coupling; within-community connections are significantly stronger than those between communities, typically representing functionally coordinated muscle units specific to the movement pattern. DEL_L: left deltoid, DEL_R: right deltoid, BB_L: left biceps brachii, BB_R: right biceps brachii, TB_L: left triceps brachii, TB_R: right triceps brachii, ECRL_L: left extensor carpi radialis longus, ECRL_R: right extensor carpi radialis longus, RF_L: left rectus femoris, RF_R: right rectus femoris, BF_L: left biceps femoris, BF_R: right biceps femoris, GC_L: left gastrocnemius, GC_R: right gastrocnemius, TA_L: left tibialis anterior, TA_R: right tibialis anterior.

Supplementary Fig. 4. Functional muscle network structure for the movement " Brush Knee and Twist Step ". Larger nodes indicate higher degree centrality, reflecting greater importance within the network, while smaller nodes indicate lower centrality. Thicker edges between nodes represent stronger coherence (higher mutual information), whereas thinner edges indicate weaker connections. "Community" refer to clusters of muscles with strong intragroup functional coupling; within-community connections are significantly stronger than those between communities, typically representing functionally coordinated muscle units specific to the movement pattern. DEL_L: left deltoid, DEL_R: right deltoid, BB_L: left biceps brachii, BB_R: right biceps brachii, TB_L: left triceps brachii, TB_R: right triceps brachii, ECRL_L: left extensor carpi radialis longus, ECRL_R: right extensor carpi radialis longus, RF_L: left rectus femoris, RF_R: right rectus femoris, BF_L: left biceps femoris, BF_R: right biceps femoris, GC_L: left gastrocnemius, GC_R: right gastrocnemius, TA_L: left tibialis anterior, TA_R: right tibialis anterior.

Supplementary Fig. 5. Functional muscle network structure for the movement " Playing the Lute ". Larger nodes indicate higher degree centrality, reflecting greater importance within the network, while smaller nodes indicate lower centrality. Thicker edges between nodes represent stronger coherence (higher mutual information), whereas thinner edges indicate weaker connections. "Community" refer to clusters of muscles with strong intragroup functional coupling; within-community connections are significantly stronger than those between communities, typically representing functionally coordinated muscle units specific to the movement pattern. DEL_L: left deltoid, DEL_R: right deltoid, BB_L: left biceps brachii, BB_R: right biceps brachii, TB_L: left triceps brachii, TB_R: right triceps brachii, ECRL_L: left extensor carpi radialis longus, ECRL_R: right extensor carpi radialis longus, RF_L: left rectus femoris, RF_R: right rectus femoris, BF_L: left biceps femoris, BF_R: right biceps femoris, GC_L: left gastrocnemius, GC_R: right gastrocnemius, TA_L: left tibialis anterior, TA_R: right tibialis anterior.

Supplementary Fig. 6. Functional muscle network structure for the movement " Repulse Monkey ". Larger nodes indicate higher degree centrality, reflecting greater importance within the network, while smaller nodes indicate lower centrality. Thicker edges between nodes represent stronger coherence (higher mutual information), whereas thinner edges indicate weaker connections. "Community" refer to clusters of muscles with strong intragroup functional coupling; within-community connections are significantly stronger than those between communities, typically representing functionally coordinated muscle units specific to the movement pattern. DEL_L: left deltoid, DEL_R: right deltoid, BB_L: left biceps brachii, BB_R: right biceps brachii, TB_L: left triceps brachii, TB_R: right triceps brachii, ECRL_L: left extensor carpi radialis longus, ECRL_R: right extensor carpi radialis longus, RF_L: left rectus femoris, RF_R: right rectus femoris, BF_L: left biceps femoris, BF_R: right biceps femoris, GC_L: left gastrocnemius, GC_R: right gastrocnemius, TA_L: left tibialis anterior, TA_R: right tibialis anterior.

Supplementary Fig. 7. Functional muscle network structure for the movement " Grasp the Bird’s Tail (Left Side) ". Larger nodes indicate higher degree centrality, reflecting greater importance within the network, while smaller nodes indicate lower centrality. Thicker edges between nodes represent stronger coherence (higher mutual information), whereas thinner edges indicate weaker connections. "Community" refer to clusters of muscles with strong intragroup functional coupling; within-community connections are significantly stronger than those between communities, typically representing functionally coordinated muscle units specific to the movement pattern. DEL_L: left deltoid, DEL_R: right deltoid, BB_L: left biceps brachii, BB_R: right biceps brachii, TB_L: left triceps brachii, TB_R: right triceps brachii, ECRL_L: left extensor carpi radialis longus, ECRL_R: right extensor carpi radialis longus, RF_L: left rectus femoris, RF_R: right rectus femoris, BF_L: left biceps femoris, BF_R: right biceps femoris, GC_L: left gastrocnemius, GC_R: right gastrocnemius, TA_L: left tibialis anterior, TA_R: right tibialis anterior.

Supplementary Fig. 8. Functional muscle network structure for the movement " Grasp the Bird’s Tail (Right Side) ". Larger nodes indicate higher degree centrality, reflecting greater importance within the network, while smaller nodes indicate lower centrality. Thicker edges between nodes represent stronger coherence (higher mutual information), whereas thinner edges indicate weaker connections. "Community" refer to clusters of muscles with strong intragroup functional coupling; within-community connections are significantly stronger than those between communities, typically representing functionally coordinated muscle units specific to the movement pattern. DEL_L: left deltoid, DEL_R: right deltoid, BB_L: left biceps brachii, BB_R: right biceps brachii, TB_L: left triceps brachii, TB_R: right triceps brachii, ECRL_L: left extensor carpi radialis longus, ECRL_R: right extensor carpi radialis longus, RF_L: left rectus femoris, RF_R: right rectus femoris, BF_L: left biceps femoris, BF_R: right biceps femoris, GC_L: left gastrocnemius, GC_R: right gastrocnemius, TA_L: left tibialis anterior, TA_R: right tibialis anterior.

Supplementary Fig. 9. Functional muscle network structure for the movement " Single Whip 1 ". Larger nodes indicate higher degree centrality, reflecting greater importance within the network, while smaller nodes indicate lower centrality. Thicker edges between nodes represent stronger coherence (higher mutual information), whereas thinner edges indicate weaker connections. "Community" refer to clusters of muscles with strong intragroup functional coupling; within-community connections are significantly stronger than those between communities, typically representing functionally coordinated muscle units specific to the movement pattern. DEL_L: left deltoid, DEL_R: right deltoid, BB_L: left biceps brachii, BB_R: right biceps brachii, TB_L: left triceps brachii, TB_R: right triceps brachii, ECRL_L: left extensor carpi radialis longus, ECRL_R: right extensor carpi radialis longus, RF_L: left rectus femoris, RF_R: right rectus femoris, BF_L: left biceps femoris, BF_R: right biceps femoris, GC_L: left gastrocnemius, GC_R: right gastrocnemius, TA_L: left tibialis anterior, TA_R: right tibialis anterior.

Supplementary Fig. 10. Functional muscle network structure for the movement " Wave Hands Like Clouds ". Larger nodes indicate higher degree centrality, reflecting greater importance within the network, while smaller nodes indicate lower centrality. Thicker edges between nodes represent stronger coherence (higher mutual information), whereas thinner edges indicate weaker connections. "Community" refer to clusters of muscles with strong intragroup functional coupling; within-community connections are significantly stronger than those between communities, typically representing functionally coordinated muscle units specific to the movement pattern. DEL_L: left deltoid, DEL_R: right deltoid, BB_L: left biceps brachii, BB_R: right biceps brachii, TB_L: left triceps brachii, TB_R: right triceps brachii, ECRL_L: left extensor carpi radialis longus, ECRL_R: right extensor carpi radialis longus, RF_L: left rectus femoris, RF_R: right rectus femoris, BF_L: left biceps femoris, BF_R: right biceps femoris, GC_L: left gastrocnemius, GC_R: right gastrocnemius, TA_L: left tibialis anterior, TA_R: right tibialis anterior.

Supplementary Fig. 11. Functional muscle network structure for the movement " Single Whip 2 ". Larger nodes indicate higher degree centrality, reflecting greater importance within the network, while smaller nodes indicate lower centrality. Thicker edges between nodes represent stronger coherence (higher mutual information), whereas thinner edges indicate weaker connections. "Community" refer to clusters of muscles with strong intragroup functional coupling; within-community connections are significantly stronger than those between communities, typically representing functionally coordinated muscle units specific to the movement pattern. DEL_L: left deltoid, DEL_R: right deltoid, BB_L: left biceps brachii, BB_R: right biceps brachii, TB_L: left triceps brachii, TB_R: right triceps brachii, ECRL_L: left extensor carpi radialis longus, ECRL_R: right extensor carpi radialis longus, RF_L: left rectus femoris, RF_R: right rectus femoris, BF_L: left biceps femoris, BF_R: right biceps femoris, GC_L: left gastrocnemius, GC_R: right gastrocnemius, TA_L: left tibialis anterior, TA_R: right tibialis anterior.

Supplementary Fig. 12. Functional muscle network structure for the movement " High Pat on Horse ". Larger nodes indicate higher degree centrality, reflecting greater importance within the network, while smaller nodes indicate lower centrality. Thicker edges between nodes represent stronger coherence (higher mutual information), whereas thinner edges indicate weaker connections. "Community" refer to clusters of muscles with strong intragroup functional coupling; within-community connections are significantly stronger than those between communities, typically representing functionally coordinated muscle units specific to the movement pattern. DEL_L: left deltoid, DEL_R: right deltoid, BB_L: left biceps brachii, BB_R: right biceps brachii, TB_L: left triceps brachii, TB_R: right triceps brachii, ECRL_L: left extensor carpi radialis longus, ECRL_R: right extensor carpi radialis longus, RF_L: left rectus femoris, RF_R: right rectus femoris, BF_L: left biceps femoris, BF_R: right biceps femoris, GC_L: left gastrocnemius, GC_R: right gastrocnemius, TA_L: left tibialis anterior, TA_R: right tibialis anterior.

Supplementary Fig. 13. Functional muscle network structure for the movement " Right Heel Kick ". Larger nodes indicate higher degree centrality, reflecting greater importance within the network, while smaller nodes indicate lower centrality. Thicker edges between nodes represent stronger coherence (higher mutual information), whereas thinner edges indicate weaker connections. "Community" refer to clusters of muscles with strong intragroup functional coupling; within-community connections are significantly stronger than those between communities, typically representing functionally coordinated muscle units specific to the movement pattern. DEL_L: left deltoid, DEL_R: right deltoid, BB_L: left biceps brachii, BB_R: right biceps brachii, TB_L: left triceps brachii, TB_R: right triceps brachii, ECRL_L: left extensor carpi radialis longus, ECRL_R: right extensor carpi radialis longus, RF_L: left rectus femoris, RF_R: right rectus femoris, BF_L: left biceps femoris, BF_R: right biceps femoris, GC_L: left gastrocnemius, GC_R: right gastrocnemius, TA_L: left tibialis anterior, TA_R: right tibialis anterior.

Supplementary Fig. 14. Functional muscle network structure for the movement " Strike to Ears with Both Fists ". Larger nodes indicate higher degree centrality, reflecting greater importance within the network, while smaller nodes indicate lower centrality. Thicker edges between nodes represent stronger coherence (higher mutual information), whereas thinner edges indicate weaker connections. "Community" refer to clusters of muscles with strong intragroup functional coupling; within-community connections are significantly stronger than those between communities, typically representing functionally coordinated muscle units specific to the movement pattern. DEL_L: left deltoid, DEL_R: right deltoid, BB_L: left biceps brachii, BB_R: right biceps brachii, TB_L: left triceps brachii, TB_R: right triceps brachii, ECRL_L: left extensor carpi radialis longus, ECRL_R: right extensor carpi radialis longus, RF_L: left rectus femoris, RF_R: right rectus femoris, BF_L: left biceps femoris, BF_R: right biceps femoris, GC_L: left gastrocnemius, GC_R: right gastrocnemius, TA_L: left tibialis anterior, TA_R: right tibialis anterior.

Supplementary Fig. 15. Functional muscle network structure for the movement " Turn and Left Heel Kick ". Larger nodes indicate higher degree centrality, reflecting greater importance within the network, while smaller nodes indicate lower centrality. Thicker edges between nodes represent stronger coherence (higher mutual information), whereas thinner edges indicate weaker connections. "Community" refer to clusters of muscles with strong intragroup functional coupling; within-community connections are significantly stronger than those between communities, typically representing functionally coordinated muscle units specific to the movement pattern. DEL_L: left deltoid, DEL_R: right deltoid, BB_L: left biceps brachii, BB_R: right biceps brachii, TB_L: left triceps brachii, TB_R: right triceps brachii, ECRL_L: left extensor carpi radialis longus, ECRL_R: right extensor carpi radialis longus, RF_L: left rectus femoris, RF_R: right rectus femoris, BF_L: left biceps femoris, BF_R: right biceps femoris, GC_L: left gastrocnemius, GC_R: right gastrocnemius, TA_L: left tibialis anterior, TA_R: right tibialis anterior.

Supplementary Fig. 16. Functional muscle network structure for the movement " Left Lower Body and Stand on One Leg ". Larger nodes indicate higher degree centrality, reflecting greater importance within the network, while smaller nodes indicate lower centrality. Thicker edges between nodes represent stronger coherence (higher mutual information), whereas thinner edges indicate weaker connections. "Community" refer to clusters of muscles with strong intragroup functional coupling; within-community connections are significantly stronger than those between communities, typically representing functionally coordinated muscle units specific to the movement pattern. DEL_L: left deltoid, DEL_R: right deltoid, BB_L: left biceps brachii, BB_R: right biceps brachii, TB_L: left triceps brachii, TB_R: right triceps brachii, ECRL_L: left extensor carpi radialis longus, ECRL_R: right extensor carpi radialis longus, RF_L: left rectus femoris, RF_R: right rectus femoris, BF_L: left biceps femoris, BF_R: right biceps femoris, GC_L: left gastrocnemius, GC_R: right gastrocnemius, TA_L: left tibialis anterior, TA_R: right tibialis anterior.

Supplementary Fig. 17. Functional muscle network structure for the movement " Right Lower Body and Stand on One Leg ". Larger nodes indicate higher degree centrality, reflecting greater importance within the network, while smaller nodes indicate lower centrality. Thicker edges between nodes represent stronger coherence (higher mutual information), whereas thinner edges indicate weaker connections. "Community" refer to clusters of muscles with strong intragroup functional coupling; within-community connections are significantly stronger than those between communities, typically representing functionally coordinated muscle units specific to the movement pattern. DEL_L: left deltoid, DEL_R: right deltoid, BB_L: left biceps brachii, BB_R: right biceps brachii, TB_L: left triceps brachii, TB_R: right triceps brachii, ECRL_L: left extensor carpi radialis longus, ECRL_R: right extensor carpi radialis longus, RF_L: left rectus femoris, RF_R: right rectus femoris, BF_L: left biceps femoris, BF_R: right biceps femoris, GC_L: left gastrocnemius, GC_R: right gastrocnemius, TA_L: left tibialis anterior, TA_R: right tibialis anterior.

Supplementary Fig. 18. Functional muscle network structure for the movement " Shuttle Back and Forth ". Larger nodes indicate higher degree centrality, reflecting greater importance within the network, while smaller nodes indicate lower centrality. Thicker edges between nodes represent stronger coherence (higher mutual information), whereas thinner edges indicate weaker connections. "Community" refer to clusters of muscles with strong intragroup functional coupling; within-community connections are significantly stronger than those between communities, typically representing functionally coordinated muscle units specific to the movement pattern. DEL_L: left deltoid, DEL_R: right deltoid, BB_L: left biceps brachii, BB_R: right biceps brachii, TB_L: left triceps brachii, TB_R: right triceps brachii, ECRL_L: left extensor carpi radialis longus, ECRL_R: right extensor carpi radialis longus, RF_L: left rectus femoris, RF_R: right rectus femoris, BF_L: left biceps femoris, BF_R: right biceps femoris, GC_L: left gastrocnemius, GC_R: right gastrocnemius, TA_L: left tibialis anterior, TA_R: right tibialis anterior.

Supplementary Fig. 19. Functional muscle network structure for the movement " Needle at Sea Bottom ". Larger nodes indicate higher degree centrality, reflecting greater importance within the network, while smaller nodes indicate lower centrality. Thicker edges between nodes represent stronger coherence (higher mutual information), whereas thinner edges indicate weaker connections. "Community" refer to clusters of muscles with strong intragroup functional coupling; within-community connections are significantly stronger than those between communities, typically representing functionally coordinated muscle units specific to the movement pattern. DEL_L: left deltoid, DEL_R: right deltoid, BB_L: left biceps brachii, BB_R: right biceps brachii, TB_L: left triceps brachii, TB_R: right triceps brachii, ECRL_L: left extensor carpi radialis longus, ECRL_R: right extensor carpi radialis longus, RF_L: left rectus femoris, RF_R: right rectus femoris, BF_L: left biceps femoris, BF_R: right biceps femoris, GC_L: left gastrocnemius, GC_R: right gastrocnemius, TA_L: left tibialis anterior, TA_R: right tibialis anterior.

Supplementary Fig. 20. Functional muscle network structure for the movement " Fan Through the Back ". Larger nodes indicate higher degree centrality, reflecting greater importance within the network, while smaller nodes indicate lower centrality. Thicker edges between nodes represent stronger coherence (higher mutual information), whereas thinner edges indicate weaker connections. "Community" refer to clusters of muscles with strong intragroup functional coupling; within-community connections are significantly stronger than those between communities, typically representing functionally coordinated muscle units specific to the movement pattern. DEL_L: left deltoid, DEL_R: right deltoid, BB_L: left biceps brachii, BB_R: right biceps brachii, TB_L: left triceps brachii, TB_R: right triceps brachii, ECRL_L: left extensor carpi radialis longus, ECRL_R: right extensor carpi radialis longus, RF_L: left rectus femoris, RF_R: right rectus femoris, BF_L: left biceps femoris, BF_R: right biceps femoris, GC_L: left gastrocnemius, GC_R: right gastrocnemius, TA_L: left tibialis anterior, TA_R: right tibialis anterior.

Supplementary Fig. 21. Functional muscle network structure for the movement " Turn, Deflect, Parry, and Punch ". Larger nodes indicate higher degree centrality, reflecting greater importance within the network, while smaller nodes indicate lower centrality. Thicker edges between nodes represent stronger coherence (higher mutual information), whereas thinner edges indicate weaker connections. "Community" refer to clusters of muscles with strong intragroup functional coupling; within-community connections are significantly stronger than those between communities, typically representing functionally coordinated muscle units specific to the movement pattern. DEL_L: left deltoid, DEL_R: right deltoid, BB_L: left biceps brachii, BB_R: right biceps brachii, TB_L: left triceps brachii, TB_R: right triceps brachii, ECRL_L: left extensor carpi radialis longus, ECRL_R: right extensor carpi radialis longus, RF_L: left rectus femoris, RF_R: right rectus femoris, BF_L: left biceps femoris, BF_R: right biceps femoris, GC_L: left gastrocnemius, GC_R: right gastrocnemius, TA_L: left tibialis anterior, TA_R: right tibialis anterior.

Supplementary Fig. 22. Functional muscle network structure for the movement " Apparent Closing Up ". Larger nodes indicate higher degree centrality, reflecting greater importance within the network, while smaller nodes indicate lower centrality. Thicker edges between nodes represent stronger coherence (higher mutual information), whereas thinner edges indicate weaker connections. "Community" refer to clusters of muscles with strong intragroup functional coupling; within-community connections are significantly stronger than those between communities, typically representing functionally coordinated muscle units specific to the movement pattern. DEL_L: left deltoid, DEL_R: right deltoid, BB_L: left biceps brachii, BB_R: right biceps brachii, TB_L: left triceps brachii, TB_R: right triceps brachii, ECRL_L: left extensor carpi radialis longus, ECRL_R: right extensor carpi radialis longus, RF_L: left rectus femoris, RF_R: right rectus femoris, BF_L: left biceps femoris, BF_R: right biceps femoris, GC_L: left gastrocnemius, GC_R: right gastrocnemius, TA_L: left tibialis anterior, TA_R: right tibialis anterior.

Supplementary Fig. 23. Functional muscle network structure for the movement " Cross Hands ". Larger nodes indicate higher degree centrality, reflecting greater importance within the network, while smaller nodes indicate lower centrality. Thicker edges between nodes represent stronger coherence (higher mutual information), whereas thinner edges indicate weaker connections. "Community" refer to clusters of muscles with strong intragroup functional coupling; within-community connections are significantly stronger than those between communities, typically representing functionally coordinated muscle units specific to the movement pattern. DEL_L: left deltoid, DEL_R: right deltoid, BB_L: left biceps brachii, BB_R: right biceps brachii, TB_L: left triceps brachii, TB_R: right triceps brachii, ECRL_L: left extensor carpi radialis longus, ECRL_R: right extensor carpi radialis longus, RF_L: left rectus femoris, RF_R: right rectus femoris, BF_L: left biceps femoris, BF_R: right biceps femoris, GC_L: left gastrocnemius, GC_R: right gastrocnemius, TA_L: left tibialis anterior, TA_R: right tibialis anterior.

Supplementary Fig. 24. Functional muscle network structure for the movement " Closing Form ". Larger nodes indicate higher degree centrality, reflecting greater importance within the network, while smaller nodes indicate lower centrality. Thicker edges between nodes represent stronger coherence (higher mutual information), whereas thinner edges indicate weaker connections. "Community" refer to clusters of muscles with strong intragroup functional coupling; within-community connections are significantly stronger than those between communities, typically representing functionally coordinated muscle units specific to the movement pattern. DEL_L: left deltoid, DEL_R: right deltoid, BB_L: left biceps brachii, BB_R: right biceps brachii, TB_L: left triceps brachii, TB_R: right triceps brachii, ECRL_L: left extensor carpi radialis longus, ECRL_R: right extensor carpi radialis longus, RF_L: left rectus femoris, RF_R: right rectus femoris, BF_L: left biceps femoris, BF_R: right biceps femoris, GC_L: left gastrocnemius, GC_R: right gastrocnemius, TA_L: left tibialis anterior, TA_R: right tibialis anterior.
